# Effectiveness of Telemedicine Interventions on Motor and Nonmotor Outcomes in Parkinson Disease: Systematic Review and Network Meta-Analysis

**DOI:** 10.2196/71169

**Published:** 2025-06-03

**Authors:** Jiejie Dou, Junyu Wang, Xianqi Gao, Guotuan Wang, Ying Bai, Yixin Liang, Kunyi Yang, Yong Yang, Lin Zhang

**Affiliations:** 1 College of Education Lishui University Lishui China; 2 School of Exercise and Health Shanghai University of Sport Shanghai China; 3 Department of Medicine Lishui University Lishui China; 4 Laboratory of Kinesiology and Rehabilitation School of Physical Education and Sport Henan University Kaifeng China; 5 Faculty of Physical Education National Research Tomsk State University Tomsk Oblast Russian Federation; 6 The School of Physical Education Kunsan National University Jeollabuk-do Republic of Korea; 7 Department of Rehabilitation West China Hospital Sichuan University Jintang Hospital Chengdu China

**Keywords:** Parkinson disease, telemedicine, e-Exercise, cognitive, meta-analysis, motor symptoms, cognitive function, quality of life

## Abstract

**Background:**

Parkinson disease (PD) presents motor and nonmotor challenges that significantly affect quality of life. Telemedicine has emerged as a promising approach to deliver interventions, including exercise performed through remote equipment (e-Exercise), cognitive behavioral training sessions conducted remotely (e-Cognitive), and consultations conducted through remote devices (e-Visits), yet their comparative effectiveness remains unclear.

**Objective:**

This paper aimed to evaluate the effectiveness of telemedicine interventions on motor and nonmotor outcomes in PD and compare the efficacy of e-Exercise, e-Cognitive, and e-Visits.

**Methods:**

A systematic review and network meta-analysis were conducted by searching PubMed, MEDLINE, Embase, Cochrane CENTRAL, and Web of Science through November 2024. Randomized controlled trials comparing telemedicine interventions with usual care were included. Outcomes assessed included total motor symptoms, quality of life, cognitive function, depressive and anxiety symptoms, fear of falling, 6-minute walk test, walking velocity, balance ability, and timed up and go. Two investigators independently performed study selection, data extraction, and risk-of-bias assessment using the Cochrane risk of bias 2 tool. Data synthesis included (1) pairwise meta-analyses using random-effects models to calculate standardized mean differences (SMDs) and mean differences; and (2) Bayesian network meta-analysis integrating direct and indirect comparisons to rank intervention efficacy, with transitivity and inconsistency evaluated. Evidence quality was graded using GRADE (Grading of Recommendations, Assessment, Development and Evaluation), incorporating risk of bias, heterogeneity (*I*²>50% indicating substantial heterogeneity), precision, and publication bias (Egger test). Statistical heterogeneity was quantified by τ² and *I*².

**Results:**

A total of 23 studies involving 1330 participants were included. Pairwise meta-analyses demonstrated that telemedicine significantly improved total motor symptoms (SMD=–0.61, 95% CI –1.19 to –0.4), cognitive function (SMD=0.58, 95% CI 0.15-1.01), depressive symptoms (SMD=–0.46, 95% CI –0.88 to –0.04), anxiety symptoms (SMD=–0.57, 95% CI –1.10 to –0.03), fear of falling (SMD=–0.48, 95% CI –0.77 to –0.19), and 6-minute walk test performance (mean difference=18.98, 95% CI 16.06-21.90 meters). The network meta-analysis revealed that e-Exercise was most effective for improving total motor symptoms (SMD=–1.01, 95% credible interval [CrI] –1.96 to –0.05) and 6-minute walk test performance. e-Cognitive was most effective for enhancing quality of life (SMD=0.39, 95% CrI 0.06-0.73) and cognitive function (SMD=1.02, 95% CrI 0.38-1.66), and reducing depressive (SMD=–1.28, 95% CrI –1.61 to –0.96) and anxiety symptoms (SMD=–1.07, 95% CrI –1.40 to –0.75). e-Visits had a limited impact across outcomes. Evidence quality was moderate or high for motor symptoms, quality of life, and depression, but low or very low for other outcomes.

**Conclusions:**

Telemedicine is effective for improving motor and nonmotor outcomes in PD. e-Exercise is optimal for motor function and physical performance, while e-Cognitive is most effective for psychological and cognitive challenges. These findings highlight the importance of tailoring telemedicine programs to address specific therapeutic needs in PD management.

**Trial Registration:**

PROSPERO CRD42024628687; https://www.crd.york.ac.uk/PROSPERO/view/CRD42024628687

## Introduction

Parkinson disease (PD) is a chronic, progressive neurodegenerative disorder characterized by the degeneration of dopaminergic neurons in the substantia nigra pars compacta, resulting in a hallmark triad of motor symptoms—bradykinesia, rigidity, and tremor—as well as a broad range of nonmotor manifestations such as cognitive impairment, mood disturbances, sleep disorders, and autonomic dysfunction [1,2]. Beyond its debilitating clinical features, PD imposes substantial morbidity and mortality, leading to functional decline and diminished independence in daily activities [3]. This progressive loss of function not only adversely affects the patients’ quality of life but also translates into a significant economic and social burden, with escalating health care costs, increased need for long-term care, and substantial caregiver strain [4,5]. Globally, the prevalence of PD continues to rise with population aging, underscoring the urgency for effective management strategies [6]. Although motor symptoms have historically defined the disease, nonmotor symptoms are increasingly recognized as pivotal determinants of disability and disease progression [7]. Improving both motor and nonmotor outcomes is, therefore, critical, as it can enhance independence, promote psychological well-being, and optimize overall health-related quality of life for individuals living with PD.

Effective PD management relies on a comprehensive, multidisciplinary approach that addresses the full spectrum of clinical manifestations, encompassing not only pharmacological therapies but also behavioral and rehabilitative interventions [8,9]. Traditional, face-to-face treatment modalities—such as cognitive behavioral therapy (CBT) for mood and cognition, structured exercise regimens to support motor function, and routine clinical visits for ongoing assessment and treatment optimization—have demonstrated efficacy in improving symptoms and quality of life [10-12]. However, these conventional approaches are limited by barriers including restricted access to specialized care, geographical distance, transportation challenges, and scheduling difficulties. These constraints often hinder patient engagement and long-term adherence, potentially diminishing therapeutic effectiveness [13]. In this context, telemedicine—the remote provision of healthcare services through electronic communication technologies—has emerged as a promising avenue to overcome traditional constraints and enhance patient-centered care in PD.

Telemedicine may improve clinical outcomes through several key mechanisms. First, eliminating geographical barriers and enabling remote consultations enhances accessibility to specialized care for underserved populations, particularly those in rural or mobility-limited settings [14]. Second, digital platforms can increase patient engagement through personalized, interactive interventions, such as remote cognitive behavioral training, gamified exercise programs, or self-monitoring tools, which integrate treatment into daily routines to promote adherence and satisfaction [15,16]. Third, continuous remote monitoring by wearable sensors or mobile apps allows clinicians to track symptom fluctuations in real-world environments, facilitating data-driven adjustments to treatment plans [17]. Telemedicine interventions for PD encompass a broad range of strategies, from remote cognitive behavioral training and exercise programs delivered through digital platforms to virtual consultations and clinical assessments [18,19]. These modalities offer the potential to deliver tailored, patient-specific interventions directly into the home environment, facilitating more frequent monitoring and timely adjustments while reducing the logistical and financial burdens of in-person visits [20]. However, the implementation of telemedicine is not without challenges. Older adults with PD may face barriers such as limited digital literacy, difficulty navigating complex technologies, or lack of access to reliable internet and devices, which could exclude vulnerable subgroups from benefiting fully [21]. In addition, patient preferences for in-person interactions or resistance to adopting new technologies may influence engagement and adherence [22,23]. Nevertheless, despite an expanding literature base, the comparative efficacy of different telemedicine modalities remains unclear. Many studies are limited by small sample sizes, heterogeneous outcome measures, and variable intervention protocols, leading to uncertainty regarding which telemedicine approaches yield the most significant and sustained clinical benefits [24,25]. This uncertainty underscores the necessity for a rigorous, systematic synthesis of the current evidence to guide clinical decision-making and inform the design of future interventions.

Against this backdrop, the primary objective of this systematic review and network meta-analysis was to evaluate the effectiveness of distinct telemedicine interventions in improving both motor and nonmotor symptoms among older adults with PD. By comparing these telemedicine strategies to each other and to usual care (UC), our aim was to identify the most efficacious remote delivery methods. We hypothesized that telemedicine interventions incorporating real-time monitoring and interactive components (eg, wearable sensors combined with gamified exercise) would demonstrate superior efficacy compared to passive remote consultations or UC. Furthermore, we sought to address three specific research questions: (1) Does telemedicine improve motor and nonmotor symptoms in patients with PD? (2) Which telemedicine modalities are most effective for improving motor symptoms (eg, bradykinesia and rigidity) in PD? and (3) Do certain remote interventions show greater benefits for nonmotor outcomes (eg, cognitive function and depression) compared to others? Such evidence will be instrumental in refining clinical practice guidelines, informing policy makers and health care providers, and ultimately improving the quality of life for individuals living with PD.

## Methods

### Overview

This preregistered systematic review and network meta-analysis (PROSPERO CRD42024628687) adhered to the reporting guidelines outlined in the PRISMA (Preferred Reporting Items for Systematic Reviews and Meta-Analyses) checklist [26] (see Multimedia Appendix 1). Quality assessment was performed using the AMSTAR 2 tool [27], applying a rigorous and consistent approach across all criteria.

### Search Strategy

We conducted a comprehensive systematic search of PubMed, MEDLINE, Embase, Cochrane CENTRAL, and Web of Science from their inception through November 3, 2024. Section S1 in Multimedia Appendix 2 shows the detailed search strategies, including terms, dates, and methodology. In addition, we reviewed reference lists of relevant articles and reviews to identify additional studies. Title and abstract screening, as well as full-text screening, were performed independently and in duplicate by investigators. Any discrepancies were resolved through discussion or, if needed, by consulting a third author for adjudication.

### Study Selection

A total of 2 reviewers independently conducted an initial screening of all identified abstracts. We only included randomized controlled trials (RCTs) that examined the effects of telemedicine in older adults with PD. We excluded non-RCTs, including quasi-experimental studies and cohort studies, to ensure that the findings were derived from studies with the highest level of evidence, minimizing potential bias in the assessment of the effects of telemedicine interventions. RCTs are considered the gold standard for evaluating intervention effects due to their ability to minimize confounding variables and biases, providing more reliable and valid results. The interventions encompassed three types of telemedicine: (1) cognitive behavioral training sessions conducted remotely (e-Cognitive), (2) exercise performed through remote equipment (e-Exercise), and (3) consultations were conducted through remote devices (e-Visits). Comparators included an active control group (aCG) that received offline exercise or cognitive training and UC. Outcome measures assessed included motor and nonmotor symptoms (Section S2 in Multimedia Appendix 2).

### Data Extraction

After retrieving all relevant articles from the specified databases, we cataloged them in EndNote X9 (Clarivate). A total of 2 authors independently extracted data from studies that met the inclusion criteria, resolving any discrepancies through consensus among all authors. Extracted data included publication details (eg, author, title, and year), patient numbers, demographics (such as age and sex), interventions, and outcome measures. If the original study reported standard errors for the experimental and control groups, we calculated the SD using the formula: SD=SE×√n. When neither SEs nor SDs were available, SDs were estimated using CIs, *t* values, quartiles, ranges, or *P* values, following the methods outlined in section 7.7.3 of the *Cochrane Handbook for Systematic Reviews*. If essential data could not be obtained through these methods, we attempted to contact the study authors up to 4 times over a 6-week period to request the required information.

### Risk of Bias

A total of 2 authors evaluated the risk of bias in the studies using the revised Cochrane risk of bias 2 tool at the study level [28], assessing the following domains: randomization process, deviations from intended interventions, missing outcome data, measurement of the outcome, and selection of the reported result. Any disagreements in the assessment were resolved by consulting a third reviewer.

### Quality of the Evidence

A total of 2 independent reviewers assessed the strength of evidence for each outcome using the GRADE (Grading of Recommendations Assessment, Development, and Evaluation) method [29]. Initially, evidence was rated as high quality and was downgraded based on the following criteria: (1) presence of publication bias (with at least 10 studies and an Egger test *P*<.05 [30]), (2) imprecision when the meta-analysis included fewer than 400 participants [31], (3) high risk of bias in more than 25% of the included studies [32], and (4) substantial heterogeneity (*I*^2^>50%) [33]. Judgments were summarized across domains into the 4 GRADE confidence levels: very low, low, moderate, or high [34].

### Data Synthesis

#### Pairwise Meta-Analysis

To determine the effectiveness of eHealth on motor and nonmotor symptoms in PD, we applied Hedges *g* of the random effects model to calculate the between-subject standardized mean difference (SMD_bs_)



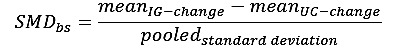



We adjusted the SMD_bs_ for the respective sample size by using the term 
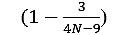
 [35] 

where N is the total sample size of the intervention group and UC, and the included studies were weighted by the SE: 
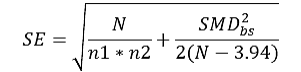
 [36], where n1 is the sample size of the intervention group and n2 is the sample size of the UC. The amount of baseline and ending change of the intervention group and UC was calculated by the following formula: *Mean_change_* = *Mean_ending_* − *Mean_baseline_*; 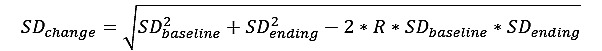 where R is a constant (R=0.5) [37]. The Cohen criteria were used to interpret the magnitude of SMD_bs_: <0.5, small; 0.5 to 0.8, moderate; and >0.8, large [38]. In addition, since the measurement units for the 6-minute walk test and timed up and go (TUG) are consistent, we calculated the mean difference.

#### Network Meta-Analysis

We used a Bayesian arm-based network meta-analysis to better understand the probability of heterogeneity between studies and to infer the probability of ranking efficacy among treatment arms. This approach provides a more accurate depiction of the likelihood of treatment ranking. We used the R (R Foundation for Statistical Computing) packages *gemtc* and *rjags* to perform network meta-analysis combining direct and indirect comparisons in the Bayesian hierarchical model [39]. A random-effects model was used to combine data, and the surface under the curved cumulative ranking probabilities was used to rank the treatments (Section S3 in Multimedia Appendix 2). The transitivity assumption was evaluated by comparing the distribution of potential effect modifiers (mean age, years of diagnosis, Hoehn and Yahr stages, sample size, percentage of male participants, and specific intervention variables) among studies grouped by comparison (Section S4 in Multimedia Appendix 2). The potential reasons for heterogeneity (mean age, years of diagnosis, Hoehn and Yahr stages, sample size, percentage of male participants, and specific intervention variables) will be explored by network meta-regression using the R *gemtc* package (Section S10 in Multimedia Appendix 2). We statistically assessed inconsistency through the design-by-treatment test for global consistency (Section S6 in Multimedia Appendix 2) [40]. In addition, since the 6-minute walk test, walking speed, balance ability, and TUG only evaluated the effects of e-Exercise on them, network meta-analysis was not performed on the above indicators.

#### Evaluation of Heterogeneity, Publication Bias, and Sensitivity Analysis

We use the tau square (τ^2^) test and the *P* value to qualitatively analyze the statistical heterogeneity between the studies. The larger the τ^2^ and the smaller the *P* value, the greater the possibility of heterogeneity; on the contrary, the smaller the existence of heterogeneity. In addition, *I*^2^ is a parameter for the quantitative analysis of the heterogeneity between the results of each study. Its value is distributed from 0% to 100%. When *I*^2^ is less than 25%, it means that the heterogeneity is low; 25% to 50% means that the heterogeneity is moderate; *I*^2^>75% means high heterogeneity. In summary, when *I*^2^>50%, it means that there is substantial heterogeneity. In addition, the Egger test is suggestive of publication bias when *P*<.05. We assessed the sensitivity of our findings by repeating each pairwise and network meta-analysis after excluding studies at overall high risk of bias (Section S7 in Multimedia Appendix 2).

### Statistical Software

All statistical analyses were conducted using R statistical software (version 4.4.1) from the R Core Team and RStudio (version 2024.04.2) from Posit. The packages involved are *metafor*, *rjags*, *gemtc*, *netmeta*, *ggplot2*, and *forestplot*.

## Results

**Figure 1:**
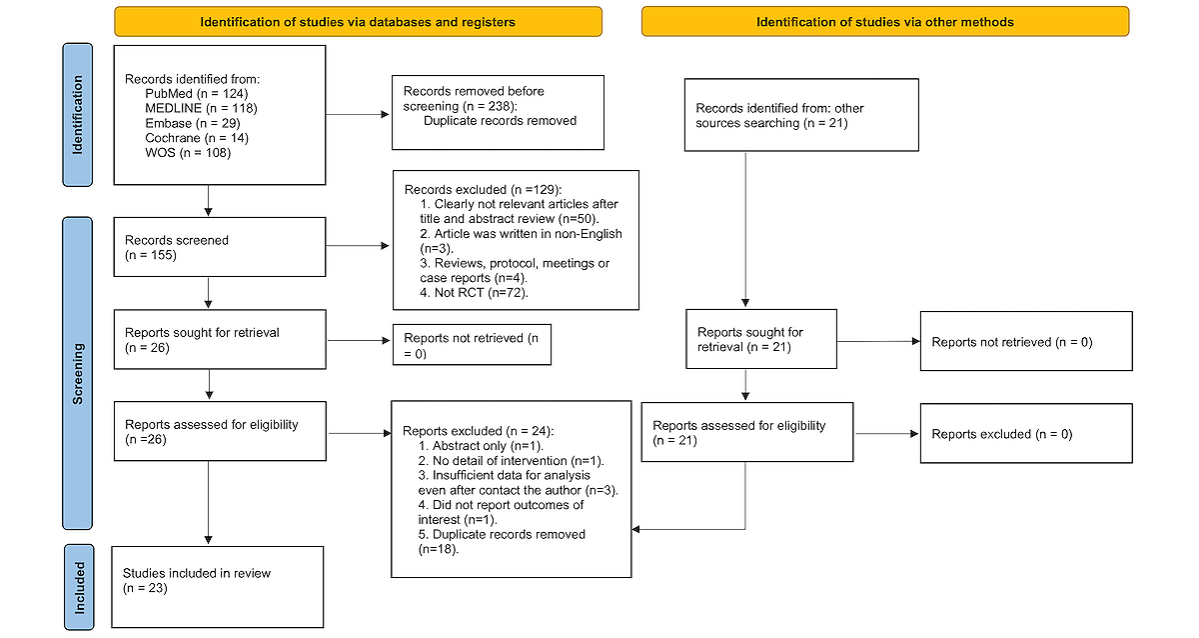
PRISMA (Preferred Reporting Items for Systematic reviews and Meta-Analyses) flow diagram of the search process for studies. RCT: randomized controlled trial; WOS: Web of Science.

Overall, 414 records were identified through the initial electronic searches. After removing duplicates, 176 records were screened for titles and abstracts, and 47 full-text articles were screened for eligibility. In total, 23 studies involving 1330 participants were included in the review (see Figure 1).

### Characteristics of Included Studies

Table 1 and Section S9 in Multimedia Appendix 2 shows the characteristics of the included studies. The year of publication ranged from 2010 to 2024. The sample size of the included studies ranged from 4 to 107. The mean age ranged from 57.87 to 72.1 years. The year of diagnosis of the included study participants ranges from 3 to 11.67 years. The Hoehn and Yahr stage ranges from 1.9 to 3. In addition, the percentage of male participants in the included studies ranged from 25% to 100%. For specific intervention variables, the range of period was 3 to 48, and the frequency was 1 to 7. The results of transitivity evaluation showed that only different types of eHealth intervention periods showed statistical differences (Section S4 in Multimedia Appendix 2). A total of 145 participants across 6 studies underwent e-Cognitive [15,41-45], 269 participants across 10 studies underwent e-Exercise [16,46-54], 255 participants across 7 studies underwent e-Visits [55-61], and 661 participants across 23 studies received UC.

**Table 1:** Characteristics of eligible randomized controlled trials (RCTs) included in the network meta-analysis.

Study and group			Age (years), mean (SD)		Sample size, n (female+male)		H&Y^a^ stage (1-5), mean (SD)		Duration of PD^b^ (years), mean (SD)		Daily LED^c^ (mg), mean (SD)		On^d^ or off^e^		Mobile health intervention details
**Park et al [41]^f^**															
	IG^g^	62.20 (7.43)		20 (15+5)		3 (0.65)		9.95 (5.26)		983.32 (352.44)		On		Based on the information-motivation-behavioral skills model; comprehensively on the information, motivation, and behavioral skill factors associated with the performance of health-related behavior; 16 weeks, daily.	
	UC^h^	64.27 (8.28)		23 (15+8)		3 (0.74)		10.50 (4.58)		916.65 (4.30)		On		Routine care via monthly phone calls or text messages.	
**Maggio et al [42]^i^**															
	IG1	59.7 (9.7)		12 (4+8)		<2.5		4.8 (2.5)		373.4 (177.0)		Off		Based on cognitive apps, and focused on concentration, spatial thinking, and reasoning skills.	
	IG2	63.8 (8.3)		12 (6+6)		<2.5		5.4 (1.9)		280.2 (126.1)		Off		Based on cognitive apps and combined with virtual reality, and focused on concentration, spatial thinking, and reasoning skills; 6 weeks, 3 times/week	
	aCG^j^	64.27 (8.28)		10 (1+9)		<2.5		3.5 (2.2)		450.0 (191.5)		Off		Home-based traditional cognitive exercises, including puzzles and memory tasks	
**Wuthrich and Rapee [43]^i^**															
	IG	68.82 (9.35)		5 (—^k^)		—		—		—		—		Telephone-delivered cognitive behavioral therapy, 10 weeks, 1 time/week	
	CG^l^	68.82 (9.35)		4 (—)		—		—		—		—		Usual care	
**Dobkin et al [15]^f^**															
	IG	67.27 (7.79)		45 (0+45)		—		5.4 (5.01)		Stable medication		On		Based on video-to-home cognitive behavioral therapy (60 min), 10 weeks, 1 time/week	
	UC	66.42 (9.51)		45 (0+45)		—		5.24 (5.13)		Stable medication		On		Usual care	
**Dobkin et al [44]^f^**															
	IG	65.62 (9.76)		37 (20+17)		—		6.95 (7.82)		—		On		Telephone-based cognitive behavioral therapy and usual care, with modules focusing on behavioral activation, cognitive restructuring, anxiety management, and sleep hygiene, 3 months, 1 time/week	
	aCG	64.80 (9.62)		35 (17+18)		—		5.65 (4.20)		—		On		Receiving psychotherapy in the community	
**Patel et al [45]^f^**															
	IG	63.1 (6.8)		14 (3+11)		—		—		Stable medication		On		A 6-week, web-based course consisting of daily “lessons” providing learnable skills and appropriate recommendations to help patients improve their sleep habits and patterns; 6 weeks, daily	
	aCG	64.7 (9.5)		14 (9+5)		—		—		Stable medication		On		Sleep hygiene advice	
**Eldemir et al [46]^i^**															
	IG	57.87 (9.79)		15 (5+10)		1.9 (0.72)		3.0 (0.98)		—		On		Based on videoconference, mainly involves manual dexterity and upper extremity exercise; 6 weeks, 3 times/week	
	aCG	61.40 (7.29)		15 (6+9)		2.0 (0.63)		4.0 (2.0)		—		On		Home-based exercise program aimed at improving balance, gait, and mobility	
**van der Kolk et al [16]^i^**															
	IG	59.3 (8.3)		65 (23+42)		1.94 (0.32)		3.42 (4.4)		600 (381.5)		On		Based on videoconference, mainly involves aerobic exercise (30 min); 6 months, 3 times/week	
	aCG	59.4 (9.3)		65 (27+38)		1.95 (0.38)		3.17 (3.8)		532 (398.5)		On		Included stretching, flexibility, and relaxation exercises (30 min)	
**Kaya Aytutuldu et al [47]^i^**															
	IG	58.47 (8.76)		16 (4+12)		2.08 (0.50)		4.82 (3.79)		761.58 (404.63)		On		Performed the LSVT BIG protocol based on synchronized telerehabilitation (60 min); 4 weeks, 4 times/week	
	aCG	61.17 (6.93)		16 (4+12)		2.02 (0.48)		6.64 (4.24)		819.58 (440.45)		On		Structured mobility-oriented exercises, cognitive tasks, and opening and closing the fists (60 min)	
**Gandolfi et al [48]^i^**															
	IG	67.45 (7.18)		38 (15+23)		2.50 (0.30)		6.16 (3.81)		—		On		Based on videoconference and combined with virtual reality, mainly involves balance exercise (50 min); 7 weeks, 3 times/week	
	aCG	69.84 (9.41)		38 (10+28)		2.50 (0.37)		7.47 (3.90)		—		On		In-clinic sensory integration balance training (50 min)	
**Vasconcellos et al [49]^i^**															
	IG	66.0 (6.3)		14 (4+10)		2.7 (0.7)		6.1 (4.4)		—		On		Based on videoconference, mainly involves trunk exercise; 3 weeks, 3 times/week	
	aCG	65.4 (10.1)		14 (6+8)		2.7 (0.6)		6.0 (4.7)		—		On		Home-based exercise program aimed at improving balance, gait, and mobility	
**Goffredo et al [50]^i^**															
	IG	67.8 (6.6)		49 (22+27)		2.0 (0.37)		4.0 (4.4)		—		On		Nonimmersive virtual reality–based telerehabilitation mainly involves balance exercise (45 min); 6-10 weeks, 3-5 times/week	
	aCG	68.2 (5.8)		48 (24+24)		2.0 (0.74)		5.0 (5.4)		—		On		Home self-administered structured conventional exercises mainly involve balance exercise (45 min)	
**Ginis et al [51]^i^**															
	IG	67.3 (8.13)		20 (3+17)		2.3 (0.4)		10.65 (5.39)		Stable medication		On		Home-based smartphone-delivered automated feedback training (balance training); 6 weeks, 3 times/week	
	aCG	66.11 (8.07)		18 (5+13)		2.2 (0.4)		11.67 (7.63)		Stable medication		On		Received weekly visits and automated feedback training (balance training)	
**Lai et al [52]^i^**															
	IG	63.4 (10.4)		10 (3+7)		2.15 (0.47)		6.55 (5.52)		Stable medication		On		Received one-on-one exercise training through the telehealth system, mainly involving 40-60% heart rate reserve (HRR) aerobic and 2-3 sets of 10-20 reps strength exercises (165 min/week); 8 weeks, 3 times/week	
	aCG	70.8 (7.1)		10 (3+7)		2.3 (0.63)		7.55 (4.78)		Stable medication		On		Home self-administered structured conventional exercises mainly involve 40-60% HRR aerobic and 2-3 sets of 10-20 reps strength exercises (165 min/week)	
**Ellis et al [53]^i^**															
	IG	64.8 (8.5)		26 (11+15)		2.0 (0.3)		5.9 (3.5)		Stable medication		On		mHealth-Supported Exercise mainly involves strength exercise (5-7 times/week) and walking (5000–7500 or 7500–10,000 per day); 12 months, not available	
	aCG	63.3 (10.6)		25 (12+13)		2.0 (0.4)		3.7 (2.1)		Stable medication		On		Strength exercise (5-7 times/week) and walking (5000-7500 or 7500-10,000 per day)	
**Khalil et al [54]^i^**															
	IG	58.4 (13.5)		16 (4+12)		2.4 (0.72)		8.0 (6.4)		—		On		The home use of an exercise DVD, walking program, and initial instructional sessions and weekly phone calls provided by a physiotherapist (45 min, reps per exercise 3-4); 8 weeks, 2 times/week	
	UC	60.7 (15.4)		14 (7+7)		2.2 (0.8)		7.5 (4.0)		—		On		Usual care	
**Dorsey et al [55]^m^**															
	IG	72.1 (7.5)		10 (3+7)		2.85 (0.70)		—		—		On		Telemedicine visits included routine clinical assessment (30-45 min, 3 times visits); 6 months, not available	
	UC	69.8 (7.0)		4 (0+4)		2.80 (0.5)		—		—		On		Usual care	
**Beck et al [56]^m^**															
	IG	65.9 (7.8)		97 (49+48)		—		8.3 (6.15)		—		On		4 virtual visits; 12 months, not available	
	CG	66.9 (8.5)		98 (42+56)		—		7.6 (4.9)		—		On		Usual care	
**Lakshminarayana et al [57]^m^**															
	IG	59.86 (9.13)		94 (34+60)		—		5.47 (4.18)		Stable medication		On		Smartphone-based Parkinson tracker app; 16 weeks, daily	
	UC	60.71 (10.26)		107 (45+62)		—		5.47 (4.89)		Stable medication		On		Usual care	
**Heldman et al [58]^m^**															
	IG	65.2 (10.1)		9 (4+5)		—		4.8 (3.5)		601 (310)		On		Telehealth management of PD using wearable sensors with videoconference or phone; 7 months, 1 time/week	
	UC	68.6 (10.2)		9 (2+7)		—		5.9 (3.7)		858 (392)		On		Usual care	
**Cubo et al [59]^m^**															
	IG	66.44 (7.09)		18 (8+10)		—		—		1143.7 (4.17)		On		Home-based motor monitoring by using wireless motion sensor technology with a phone; 1 year, 1 time/week	
	UC	66.05 (9.76)		20 (12+8)		—		—		133.4 (518.8)		On		Usual care	
**Dorsey et al [60]^m^**															
	IG	66.6 (12.0)		9 (2+7)		2.3 (0.6)		—		—		On		Telemedicine visits included routine clinical assessment (4 times visits); 7 months, not available	
	UC	64.5 (11.3)		11 (3+8)		2.5 (1.0)		—		—		On		Usual care	
**Wilkinson et al [61]^m^**															
	IG	67.2 (9.8)		18 (—)		2.5 (1.1)		—		—		On		Clinical video telehealth; 12 months, not available	
	UC	70.9 (8.4)		18 (—)		2.3 (0.4)		—		—		On		Usual care	

^a^H&Y stage: Hoehn and Yahr Scale.

^b^PD: Parkinson disease.

^c^LED: levodopa equivalent dose.

^d^On: 1-2 hours after taking their normal dopaminergic medication.

^e^Off: >12 hours withdrawal from dopaminergic medication.

^f^Cognitive behavioral training based on telemedicine.

^g^IG: intervention group.

^h^UC: usual care.

^i^Telemedicine-based exercise.

^j^aCG: activity control group.

^k^N/A: not available.

^l^CG: control group.

^m^Telemedicine visits.

### Pairwise Meta-Analysis

A total of 12 SMD_bs_ in 12 (52.2%) out of 23 studies with 666 (50.1%) out of 1330 participants showed changes in total motor symptoms [16,41,46,50,51,54-56,58-61]. There were moderated effects of telemedicine in relieving total motor symptoms (SMD_bs_=–0.67, 95% CI –1.25 to –0.09; *I*^2^=90.3%; *P*=.02). A total of 16 SMD_bs_ in 16 (69.6%) out of 23 studies with 940 (70.7%) out of 1330 participants showed changes in quality of life that demonstrated small improvement (SMD_bs_=0.05, 95% CI –0.08 to 0.18; *I*^2^=12.1%; *P*=.43) [16,41,44-48,51,53,55-61]. A total of 5 SMD_bs_ in 4 (17.4%) out of 23 studies with 266 (20%) out of 1330 participants showed changes in cognitive level that demonstrated moderate improvement (SMD_bs_=0.56, 95% CI 0.14-0.97; *I*^2^=42.1%; *P*=.01) [42,51,55,56]. This also was shown for depressive (10 SMD_bs_ in 10/23, 43.5% studies with 746/1330, 56.1% participants) [15,16,43-45,55-57,59,61] and anxiety symptoms (5 SMD_bs_ in 5/23, 21.7% studies with 458/1330, 34.4% participants) [15,16,43,44,57] that demonstrated small to moderate relief (depressive symptoms: SMD_bs_=–0.45, 95% CI –0.86 to –0.03; *I*^2^=84.7%; *P*=.04; anxiety symptoms: SMD_bs_=–0.55, 95% CI –1.08 to –0.03; *I*^2^=84.7%; *P*=.04). A total of 5 SMD_bs_ in 5 (21.7%) out of 23 studies with 306 (23%) out of 1330 participants showed changes in fear of falling that demonstrated small relief (SMD_bs_=–0.46, 95% CI –0.69 to –0.23); *I*^2^=26.8%; *P*<.001) [16,47,48,51,54]. In addition, telemedicine significantly improved the 6-minute walk test compared to usual care (mean difference_bs_=18.98, 95% CI 16.10-21.86 meters; *I*^2^=0%; *P*<.001) [16,50-54]. However, walking speed [47-52,54], balance ability [16,47,48,50,51,54], and TUG [16,47,50] were not significantly improved. Even though TUG had a significant trend (*P*=.05). In addition, the results of the Egger test (*P*>.05) showed that no publication bias was found (Table 2 and Section S8 in Multimedia Appendix 2).

**Table 2:** The results of pairwise meta-analysis.

Outcome measures	Trials, n	Sample size (EX^a^+UC^b^)	SMD^c^ or MD^d^ (95% CI)	*I*^2^ (%)	*P* value	*P* value (test of effect size)	*P* value (Egger test)	Publication bias (%)	Quality of the evidence
Total motor symptoms	12	666 (335+331)	–0.67 (–1.25 to –0.09)	90.3	<.001	.02	.35	8.3	Moderate
Quality of life	16	940 (462+478)	0.05 (–0.08 to 0.18)	12.1	.32	.43	.21	12.5	High
Cognitive level	4	266 (144+122)	0.56 (0.14-0.97)	42.1	.11	.01	.05	0	Low
Depressive symptoms	10	746 (364+382)	–0.45 (–0.86 to –0.03)	84.7	<.001	.04	.34	20	Moderate
Anxiety symptoms	5	458 (220+238)	–0.55 (–1.08 to –0.03)	84.7	<.001	.04	.44	20	Low
Fear of falling	5	306 (155+151)	–0.46 (–0.69 to –0.23)	26.8	.24	<.001	.60	0	Low
6-minute walking test	6	366 (186+180)	18.98 (16.10-21.86)^e^	0	.73	<.001	.87	0	Low
Walking velocity	7	321 (163+158)	0.57 (–0.33 to 1.46)	93.4	<.001	.21	.88	0	Very low
Balance ability	6	403 (204+199)	0.69 (–0.24 to 1.62)	94.1	<.001	.15	.43	0	Low
Timed up and go	3	259 (130+129)	0.08 (–0.00 to 0.17)^e^	0	.44	.05	.55	0	Low

^a^EX: e-Exercise.

^b^UC: usual care.

^c^SMD: standardized mean difference.

^d^MD: mean difference.

^e^The effect size is the mean difference.

### Network Meta-Analysis

**Figure 2:**
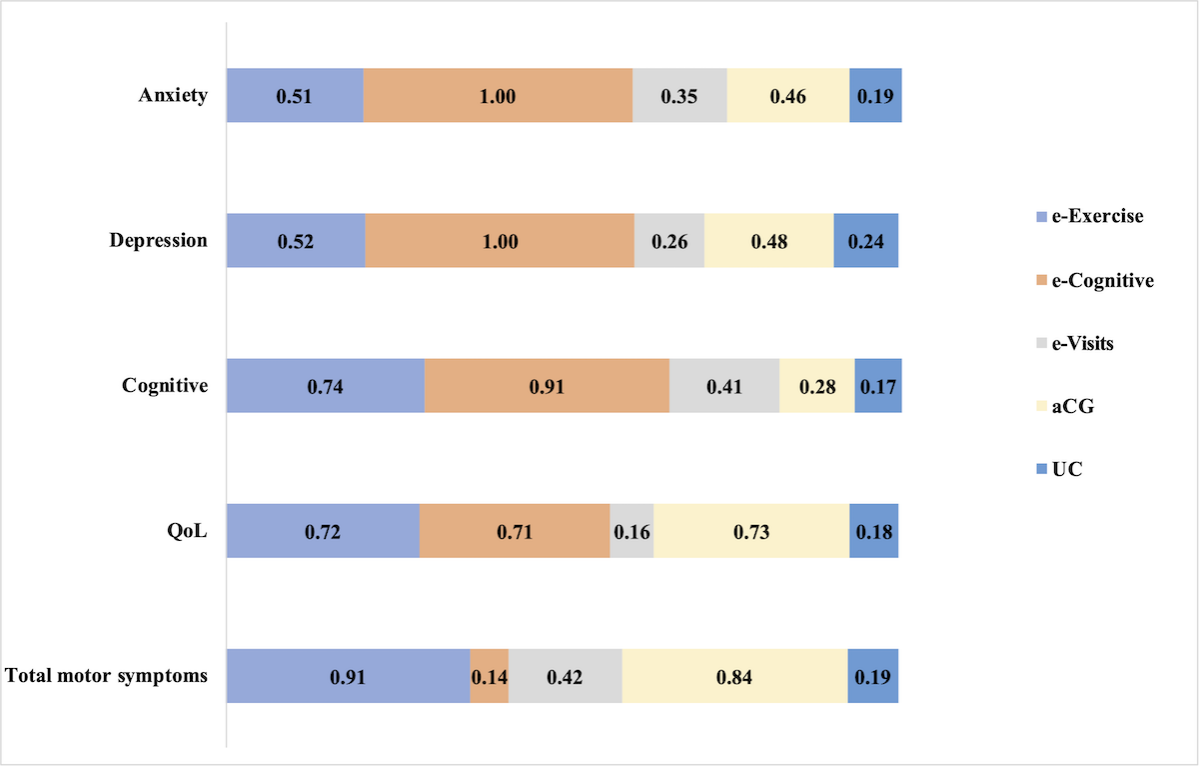
Column chart based on the surface under the curved cumulative ranking probabilities. aCG: active control group; e-Cognitive: cognitive behavioral training sessions conducted remotely; e-Exercise: exercise performed through remote equipment; e-Visits: consultations conducted through remote devices; QoL: quality of life; UC: usual care.

A total of 12 studies involving 666 participants assessed total motor symptoms. Section S5.1 in Multimedia Appendix 2 presents the direct comparisons and sample size distribution among telemedicine types for total motor symptoms. The network meta-analysis revealed that only e-Exercise (SMD=–2.38, 95% credible interval [CrI] –3.36 to –1.39) and aCG (SMD=–2.23, 95% CrI –3.48 to –0.98) significantly alleviated total motor symptoms compared to UC (Table 3). Ranking the telemedicine types by their effectiveness in reducing total motor symptoms, e-Exercise was the most effective (Figure 2; surface under the cumulative ranking curve [SUCRA] 0.91). At the same time, e-Exercise and aCG also significantly improved the overall motor symptoms of patients with PD compared with e-Cognitive and e-Visits (Section S5.1 in Multimedia Appendix 2). Heterogeneity analysis indicated a moderate level (Section S6 in Multimedia Appendix 2: *I*²=49%, τ²=0.1000, *P*=.06). The design-by-treatment interaction test showed no significant global inconsistency (Section S6 in Multimedia Appendix 2: *P*=.54).

A total of 16 studies involving 947 participants assessed quality of life. Section S5.2 in Multimedia Appendix 2 presents the direct comparisons and sample size distribution among telemedicine types for quality of life. The results of network meta-analysis showed that aCG, e-Exercise, and e-Cognitive had similar effects in improving the quality of life of patients with PD, but only e-Cognitive significantly improved the quality of life of patients with PD compared with UC, with an SMD of 0.51 (95% CrI 0.14-0.88), and was significantly superior to e-Visits (Section S5.2 in Multimedia Appendix 2). Ranking the telemedicine types by their effectiveness in improving quality of life, aCG was the most effective (Figure 2: SUCRA 0.73). Heterogeneity analysis indicated a low level (*I*²=0%, τ²=0, *P*=.61; see Section S6 in Multimedia Appendix 2). The design-by-treatment interaction test showed no significant global inconsistency (*P*=0.97; see Section S6 in Multimedia Appendix 2).

Furthermore, 4 studies involving 266 participants assessed cognitive level. Section S5.3 in Multimedia Appendix 2 presents the direct comparisons and sample size distribution among telemedicine types for the cognitive level. The network meta-analysis revealed that only e-Cognitive (SMD=1.09, 95% CrI 0.18-2.00) significantly improved cognitive level compared to UC. In addition, e-Cognitive (SMD=0.95, 95% CrI 0.06-1.84) and e-Exercise (SMD=0.68, 95% CrI 0.02-1.34) were significantly superior to aCG (Section S5.3 in Multimedia Appendix 2). Ranking the telemedicine types by their effectiveness in improving cognitive level, e-Cognitive was the most effective (Figure 2: SUCRA 0.91). Heterogeneity analysis indicated a low level (*I*²=0%; τ²=0; *P*=0.63; see Section S6 in Multimedia Appendix 2). The design-by-treatment interaction test showed no significant global inconsistency (*P*=0.42; see Section S6 in Multimedia Appendix 2).

**Table 3:** The results of network meta-analysis.

Outcome measures		Reference UC^a^		Reference aCG^b^			SUCRA^c^
		Direct comparisons, n	SMD^d^ (95% CrI^e^)	Direct comparisons, n	SMD (95% CrI)		
**Total motor symptoms**							
	e-Exercise^f^	1	–2.38 (–0.36 to –1.39)	4	–0.15 (–0.91 to 0.62)	0.91	
	aCG	0	–2.23 (–3.48 to –0.98)	—^g^	—	0.84	
	e-Visits^h^	6	–0.36 (–0.95 to 0.23)	0	1.87 (0.49-3.25)	0.42	
	UC	—	—	0	2.23 (0.98-3.48)	0.19	
	e-Cognitive^i^	1	0.25 (–1.09 to 1.59)	0	2.48 (0.64-4.31)	0.14	
**Quality of life**							
	aCG	0	0.59 (–0.24 to 1.42)	—	—	0.73	
	e-Exercise	0	0.58 (–0.27 to 1.44)	6	–0.00 (–0.21 to 0.20)	0.72	
	e-Cognitive	2	0.51 (0.14-0.88)	1	–0.08 (–0.82 to 0.66)	0.71	
	UC	—	—	0	–0.59 (–1.42 to 0.24)	0.18	
	e-Visits	7	–0.01 (–0.20 to 0.18)	0	–0.60 (–1.45 to 0.25)	0.16	
**Cognitive level**							
	e-Cognitive	1	1.09 (0.18-2.00)	1	0.95 (0.06-1.84)	0.91	
	e-Exercise	0	0.82 (–0.62 to 2.25)	1	0.68 (0.02-1.34)	0.74	
	e-Visits	2	0.18 (–0.11 to 0.46)	0	0.03 (–1.27 to 1.34)	0.41	
	aCG	0	0.14 (–1.14 to 1.42)	—	—	0.28	
	UC	—	—	0	–0.14 (–1.42 to 1.14)	0.17	
**Depressive symptoms**							
	e-Cognitive	2	–1.40 (–1.90 to –0.91)	2	–1.16 (–1.63 to –0.69)	1	
	e-Exercise	0	–0.28 (–1.08 to 0.51)	1	–0.04 (–0.46 to 0.38)	0.52	
	aCG	0	–0.24 (–0.92 to 0.44)	—	—	0.48	
	e-Visits	5	–0.01 (–0.24 to 0.23)	0	0.23 (–0.48 to 0.95)	0.26	
	UC	—	—	0	0.24 (–0.44 to 0.92)	0.24	
**Anxiety symptoms**							
	e-Cognitive	2	–1.18 (–1.61 to –0.75)	1	–0.93 (–1.43 to –0.44)	1	
	e-Exercise	0	–0.28 (–1.03 to 0.46)	1	–0.04 (–0.39 to 0.31)	0.51	
	aCG	0	–0.25 (–0.90 to 0.41)	—	—	0.46	
	e-Visits	1	–0.09 (–0.42 to 0.23)	0	0.15 (–0.58 to 0.88)	0.35	
	UC	—	—	0	0.25 (–0.41 to 0.90)	0.19	

^a^UC: usual care.

^b^aCG: active control group.

^c^SUCRA: surface under the cumulative ranking curve.

^d^SMD: standardized mean difference.

^e^CrI: credible interval.

^f^e-Exercise: exercise performed through remote equipment.

^g^Not available.

^h^e-Visits: consultations conducted through remote devices.

^i^e-Cognitive: cognitive behavioral training sessions conducted remotely.

In addition, 10 studies involving 746 participants assessed depressive symptoms. Section S5.4 in Multimedia Appendix 2 presents the direct comparisons and sample size distribution among telemedicine types for depressive symptoms. The network meta-analysis revealed that only e-Cognitive significantly alleviated depressive symptoms compared to UC, with an SMD of –1.40 (95% CrI –1.90 to –0.91), and was significantly superior to e-Exercise, aCG, and e-Visits (see Section S5.4 in Multimedia Appendix 2). Ranking the telemedicine types by their effectiveness in reducing depressive symptoms, e-Cognitive was the most effective (Figure 2: SUCRA 1.00). Heterogeneity analysis indicated a low level (*I*²=16.4%; τ²=0.0146; *P*=.30; see Section S6 in Multimedia Appendix 2). The design-by-treatment interaction test showed no significant global inconsistency (*P*=.81; see Section S6 in Multimedia Appendix 2).

A total of 5 studies involving 458 participants assessed anxiety symptoms. Section S5.5 in Multimedia Appendix 2 presents the direct comparisons and sample size distribution among telemedicine types for anxiety symptoms. The network meta-analysis revealed that only e-Cognitive significantly alleviated anxiety symptoms compared to UC, with an SMD of –1.18 (95% CrI –1.61 to –0.75), and was significantly superior to e-Exercise, aCG, and e-Visits (Section S5.5 in Multimedia Appendix 2). Ranking the telemedicine types by their effectiveness in reducing anxiety symptoms, e-Cognitive was the most effective (Figure 2: SUCRA 1.00). Heterogeneity analysis indicated a low level (*I*²=0.4%; τ²=0.0011; *P*=.32; Section S6 in Multimedia Appendix 2). The design-by-treatment interaction test showed no significant global inconsistency (*P*=.84; see Section S6 in Multimedia Appendix 2).

### Risk of Bias and Quality of the Evidence

**Figure 3:**
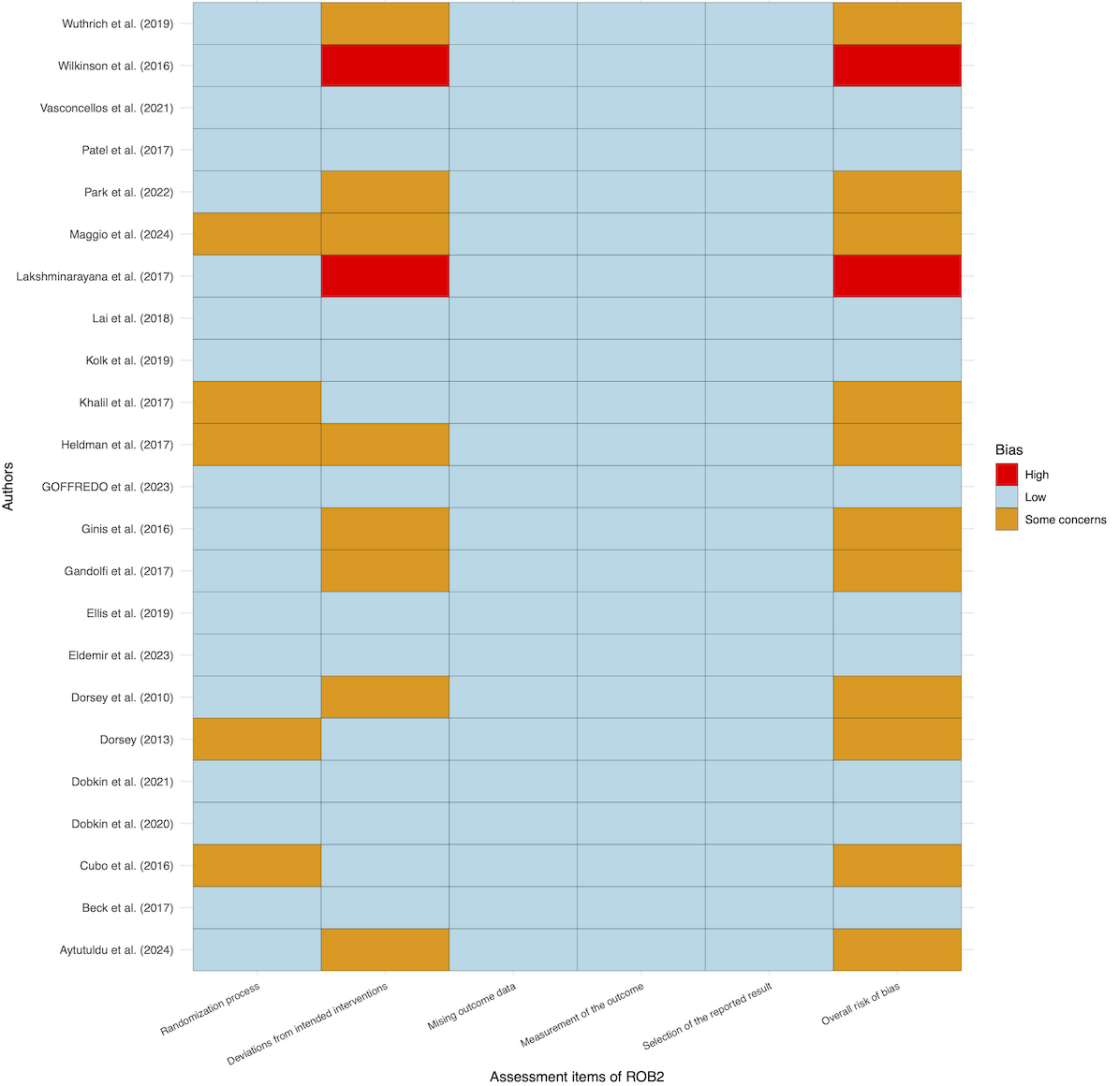
Cochrane Risk of Bias 2 (RoB2) items heat map for included studies.

Of the 23 trials, for overall bias, 10 studies were assessed as low risk of bias, 11 as some concerns, and 2 as high risk of bias. In the randomization process, 18 trials were assessed as low risk and 5 trials as some concerns; for deviations from intended interventions, 13 trials were assessed as low risk, 8 trials as some concerns, and 2 as high risk; and in the missing outcome data, the measurement of the outcome, and the selection of the reported result, all 23 trials were all low risk (Figure 3 [15,16,41-61]). Regarding the grade of evidence for the outcomes, our results showed that only total motor symptoms, quality of life, and depressive symptoms were rated moderate or high, while the other outcome indicators were rated low to very low (Table 2).

## Discussion

### Principal Findings

This meta-analysis, which included 23 RCTs and 1330 participants with PD, suggested potential benefits of telemedicine interventions that require cautious interpretation. Paired meta-analysis results showed trends toward improvements in total motor symptoms, cognitive function, depression and anxiety symptoms, fear of falling, and performance on the 6-minute walk test, but high heterogeneity (*I*^2^>75%) and small sample sizes (fewer than 400) limited the certainty of these estimates. Network meta-analysis results indicated that e-Exercise showed a relatively large effect in relieving total motor symptoms compared with other modalities, but the small number of studies (1 study for UC and 4 studies for aCG) prevented definitive conclusions about its clinical usability. Although e-Cognitive provided the greatest improvements in quality of life, cognitive performance, and psychological well-being, the small number of studies combined also limited the confidence in the results. These preliminary findings highlight methodologically limited evidence that can inform exploratory discussions about telemedicine strategies for the management of PD. Given the low certainty of evidence (GRADE assessment) and considerable heterogeneity across studies, policy makers and clinicians should consider these results as hypothesis generating rather than practice guidance, awaiting confirmation by rigorously designed trials with standardized outcome measures and reduced risk of high heterogeneity.

Telemedicine has the potential to overcome traditional barriers such as geographic isolation, transportation challenges, and limited access to specialist care, making it a promising strategy in the management of PD [62]. In our analysis, the telehealth intervention was associated with improvements in multiple motor and nonmotor outcomes, including key parameters of motor function, cognitive performance, and psychological well-being. However, given the considerable heterogeneity noted across several outcome measures and the small sample sizes, it is important to interpret these findings with caution. Previous studies have highlighted the flexibility and accessibility of telemedicine, which facilitates timely intervention, improved adherence, and more frequent patient-provider interactions [63]. Mechanistically, the ability of telemedicine to integrate tailored exercise regimens, cognitive training modules, and ongoing psychosocial support within the home environment may enhance patient engagement, motivation, and symptom self-management, thereby translating into meaningful clinical benefits [64,65]. However, the absence of significant improvements in certain outcomes, such as walking velocity, balance ability, TUG, and overall quality of life, may reflect the multifactorial and context-dependent nature of these domains. These measures often involve complex motor control, postural stability, and multidimensional psychosocial components that may require more intensive, prolonged, or multimodal interventions than those typically delivered via telemedicine [66]. In addition, variations in intervention intensity, technological interfaces, and patient-specific factors—such as disease severity, baseline functional capacity, and individual learning curves—could limit the capacity of current telemedicine protocols to produce uniform gains in these complex domains [67]. As such, while telemedicine clearly holds promise for enhancing several key outcome measures, optimizing intervention design, dosing, and implementation strategies will be essential to fully realize its potential across the broader spectrum of PD-related impairments.

Building upon the broader efficacy observed with telemedicine, these findings underscore the importance of intervention specificity, as e-Exercise, e-Cognitive, and e-Visits target distinct facets of PD management. Within this framework, this study performed a more granular analysis of these telemedicine modalities. Notably, e-Exercise emerged as particularly effective in alleviating overall motor symptoms compared with the UC group, and the effects were similar to those of the offline exercise intervention (all aCG groups in the included literature evaluating Unified Parkinson's Disease Rating Scale 3 were involved in offline exercise), a result that corroborates previous research demonstrating the potential value of exercise-based interventions in improving gait, rigidity, and bradykinesia among individuals with PD [68]. This outcome may be attributed to several interrelated mechanisms. First, e-Exercise programs often incorporate structured, progressive physical activities that can enhance muscular strength, flexibility, and aerobic capacity—key factors that positively influence postural control, stride length, and movement fluidity [69]. Second, through advanced monitoring tools and interactive feedback systems, e-Exercise platforms may facilitate greater adherence and motivation, enabling patients to practice targeted exercises at an optimal frequency and intensity [70]. Third, the neuroplastic changes associated with regular physical activity, such as improved dopaminergic signaling and synaptic efficiency, may partially counteract PD-related degenerative processes, thereby yielding more pronounced gains in overall motor function [71]. By engaging patients in a consistent and scientifically informed exercise regimen, e-Exercise provides a focused and practical approach to addressing motor deficits in PD.

Among all telemedicine approaches examined, e-Cognitive emerged as uniquely effective in improving quality of life, cognitive function, and emotional well-being in individuals with PD compared with the UC group. These interventions typically encompass elements such as remotely delivered cognitive-behavioral therapy, personalized cognitive training modules, and structured psychosocial support sessions. Such strategies can promote more adaptive neural network activity, enhance frontal-executive functioning, and stimulate neuroplastic changes, potentially mitigating the cognitive decline associated with PD-related dopaminergic and cholinergic deficits [72]. Concurrently, the cognitive-behavioral components of e-Cognitive interventions may bolster patients’ self-efficacy, reduce maladaptive thought patterns, and provide patients with practical tools to cope with both disease-specific stressors and emotional instability [10]. As patients gain greater control over their symptom perception and response, they often experience improved mood, reduced anxiety and depressive symptoms, and a stronger sense of autonomy and competence. This, in turn, can reverberate through multiple domains of daily life—improved executive functioning and psychological resilience facilitate more effective participation in social, recreational, and rehabilitative activities, cumulatively enhancing overall quality of life. These processes align with and extend previous research on cognitive-behavioral therapies in neurodegenerative conditions, suggesting that the interplay between enhanced neural adaptability, strengthened coping strategies, and enriched psychosocial support underpins the superior patient-reported outcomes observed with e-Cognitive interventions [73].

It is worth noting that aCG and e-Exercise may also be effective interventions to improve the quality of life of patients with PD. For example, our research results found that aCG and e-Exercise were ranked higher than e-Cognitive in improving the quality of life of patients with PD, even though there was no significant difference compared with the UC group. The results of Eldemir et al [46] showed that both e-Exercise and offline exercise significantly improved the quality of life of patients with PD after 18 sessions over 6 weeks. However, the results of e-Exercise and aCG compared with the UC group were from indirect comparisons, resulting in a wider 95% CrI, which resulted in them not significantly improving the quality of life of patients with PD compared with the UC group. At the same time, the same reason caused e-Exercise to not significantly improve the cognitive level of patients with PD compared with the UC group (Table 3). However, this does not negate the effectiveness of e-Exercise in alleviating the cognitive level of patients with PD. As our results found, compared with the aCG group, both e-Exercise and e-Cognitive significantly improved the cognitive level of patients with PD.

Previous studies have demonstrated logistical benefits of teleconsultation, such as reduced travel time and improved accessibility [74]. However, the relative underperformance of e-Visits compared with the favorable outcomes of e-Exercise and e-Cognition raises important questions about the clinical use of this particular telehealth model. However, it is important to clarify that the core results of this study were beneficial compared with those of the UC group. Therefore, we cannot conclude that e-Visits are an ineffective electronic intervention. As shown in the results of Beck et al [56], 12-month e-Visits not only had good compliance (98%) but also had similar benefits to UC in reducing overall motor symptoms in patients with PD. The results of Lakshminarayana et al [57] also showed that e-Visits can be an effective and novel method to improve self-reported medication adherence and clinical consultation quality by supporting self-management in PD with smartphones. However, unlike e-Exercise, which offers structured physical training, or e-Cognitive, which provides systematic cognitive-behavioral strategies, e-Visits often focus predominantly on general assessments and routine follow-ups. The absence of hands-on, protocol-driven activities or interactive training components likely limits the capacity of e-Visits to influence key motor, cognitive, or emotional outcomes [25]. In addition, variability in platform quality, the nuances of patient-provider communication, and the restricted scope of interventions delivered through e-Visits may further constrain their impact [75]. These results suggest that while e-Visits retain value for ongoing monitoring, medication management, and convenience, their contribution to improving clinical outcomes may be enhanced by integrating more structured and multidimensional telemedicine modalities.

This study has several notable strengths. First, it used a comprehensive and systematic approach, integrating data from 23 RCTs to ensure that a broad spectrum of telemedicine interventions for PD was represented. Second, the use of network meta-analysis facilitated robust indirect comparisons, enabling a more nuanced understanding of the relative effectiveness of distinct telemedicine modalities than would have been possible through conventional pairwise comparisons alone. Nevertheless, 3 important limitations must be acknowledged. First, variability in intervention protocols and outcome measures, as well as small sample sizes in some included studies, may have introduced heterogeneity that limited the precision and generalizability of the findings. Second, the absence of long-term follow-up data constrains the ability to determine whether the observed benefits of telemedicine interventions are sustained over extended periods, which is particularly relevant in a chronic, progressive disease like PD. Third, while we accounted for demographic and clinical covariates (eg, age and disease stage), our transitivity assessment was limited by incomplete reporting of intervention-specific parameters. Key modifiers such as telemedicine duration (median 10 weeks), adherence metrics (reported in only 17% of trials), and technological literacy assessments (explicitly measured in 1 study) exhibited substantial heterogeneity in measurement and reporting. This may have confounded effect estimates, particularly given that unmeasured variations in technological access could disproportionately affect outcomes in older adults with PD. Fourth, the inherent complexity of PD, including the interplay of motor and nonmotor symptoms and the influence of individual patient characteristics, poses challenges to controlling for potential confounding variables and fully elucidating the mechanisms underlying observed outcomes. Future research should address these methodological considerations to optimize telemedicine strategies and maximize their impact on PD management, particularly through standardized reporting of implementation science parameters (eg, intervention exposure duration, adherence tracking protocols, and technology accessibility metrics).

### Conclusion

This systematic review of 23 RCTs (n=1330) suggests that telehealth may benefit some PD outcomes, with e-Exercise showing greater motor improvements and e-Cognitive being associated with cognitive and psychological gains, although high heterogeneity (*I*²=82%) and small sample sizes limit certainty. Current evidence supports exploratory prioritization of these modalities, while acknowledging no effects on gait and balance and very low GRADE certainty. Rigorous trials are needed to standardize interventions, control for bias, and validate long-term efficacy (≥6 months) before clinical implementation.

## Data Availability

The datasets generated or analyzed during this study are available from the corresponding author on reasonable request.

## Appendix

Multimedia Appendix 1 Revised PRISMA (Preferred Reporting Items for Systematic Reviews and Meta-Analyses) checklist.

Multimedia Appendix 2 Supplementary materials.

Multimedia Appendix 3 We have revised the contents of the Multimedia Appendix 2 table of contents, and this is the revised version.

## Abbreviations

aCG: active control group
CBT: cognitive behavioral therapy
CrI: credible interval
e-Cognitive: cognitive behavioral training sessions conducted remotely
e-Exercise: exercise performed through remote equipment
e-Visits: consultations conducted through remote devices
GRADE: Grading of Recommendations Assessment, Development, and Evaluation
PD: Parkinson disease
RCT: randomized controlled trial
SMD: standardized mean difference
SUCRA: surface under the cumulative ranking curve.
TUG: timed up and go
UC: usual care
