# Supplementary material for: Effectiveness of Telemedicine Interventions on Motor and Nonmotor Outcomes in Parkinson Disease: Systematic Review and Network Meta-Analysis
Source: J Med Internet Res. 2025 Jun 3;27:e71169. doi: 10.2196/71169 (PMC12174881; doi:10.2196/71169)
Supplement: Multimedia Appendix 2 [file jmir_v27i1e71169_app2.docx]

**Appendix**

Table of Contents:

[Section S1: Search Strategy 3](#_Toc194567969)

[Database: PubMed <inception to September 30 2024> 3](#_Toc194567970)

[Section S2: Outcomes 5](#_Toc194567971)

[Section S3: Statistical methods in details 7](#_Toc194567972)

[Network meta-analysis 7](#_Toc194567973)

[Continuous variable-random effects model: 7](#_Toc194567974)

[Assessment of the transitivity assumption 9](#_Toc194567975)

[Assessment of heterogeneity and inconsistency 9](#_Toc194567976)

[Assessment of sensitivity 9](#_Toc194567977)

[Statistical software 9](#_Toc194567978)

[Section S4: Assessment of the transitivity 10](#_Toc194567979)

[4.1 Sample size 10](#_Toc194567980)

[4.2 Mean age 11](#_Toc194567981)

[4.3 Years of diagnosis 12](#_Toc194567982)

[4.4 Hoehn and Yahr stage 13](#_Toc194567983)

[4.5 Percentage male 14](#_Toc194567984)

[4.6 Intervention period 15](#_Toc194567985)

[4.7 Intervention frequency 16](#_Toc194567986)

[4.8 Total number of interventions 17](#_Toc194567987)

[Section S5: Network plot and league table 18](#_Toc194567988)

[5.1 Motor symptoms (UPDRS 3) 18](#_Toc194567989)

[5.2 Quality of life 22](#_Toc194567990)

[5.3 Cognitive level 26](#_Toc194567991)

[5.4 Depressive Symptoms 30](#_Toc194567992)

[5.5 Anxiety Symptoms 34](#_Toc194567993)

[Section S6: Evaluation of heterogeneity and inconsistency 38](#_Toc194567994)

[Section S7: Sensitivity analysis 39](#_Toc194567995)

[7.1 Sensitivity analysis for pairwise meta-analysis 39](#_Toc194567996)

[7.2 Sensitivity analysis for network meta-analysis 41](#_Toc194567997)

[Section S8: Pairwise meta-analysis 45](#_Toc194567998)

[Section S9: Evaluation outcome measurement method included in the literature 48](#_Toc194567999)

# SectionS1: Search Strategy

## Database: PubMed <inception to September 30 2024>

***Search Strategy:***

| **Search number** | **Query** | **Results** |
| --- | --- | --- |
| 28 | ((Parkinson disease[MeSH Terms]) AND ((((((((((((((((((Telemedicine[MeSH Terms]) OR (Virtual Medicine)) OR (Medicine, Virtual)) OR (Tele-Referral)) OR (Tele Referral)) OR (Tele-Referrals)) OR (Mobile Health)) OR (Health, Mobile)) OR (mHealth)) OR (Telehealth)) OR (eHealth)) OR (Tele-Intensive Care)) OR (Tele Intensive Care)) OR (Tele-ICU)) OR (Tele ICU)) OR (Telecare)) OR (Tele-Care)) OR (Tele Care))) AND ((((((randomized controlled trial[Publication Type]) OR (randomized[Title/Abstract])) OR (placebo[Title/Abstract])) OR (randomly[Title/Abstract])) OR (trial[Title])) OR (clinical trials as topic[MeSH Terms])) | 124 |
| 27 | (((((randomized controlled trial[Publication Type]) OR (randomized[Title/Abstract])) OR (placebo[Title/Abstract])) OR (randomly[Title/Abstract])) OR (trial[Title])) OR (clinical trials as topic[MeSH Terms]) | 1,672,993 |
| 26 | clinical trials as topic[MeSH Terms] | 398,630 |
| 25 | trial[Title] | 320,317 |
| 24 | randomly[Title/Abstract] | 445,471 |
| 23 | placebo[Title/Abstract] | 260,096 |
| 22 | randomized[Title/Abstract] | 728,192 |
| 21 | randomized controlled trial[Publication Type] | 625,224 |
| 20 | (((((((((((((((((Telemedicine[MeSH Terms]) OR (Virtual Medicine)) OR (Medicine, Virtual)) OR (Tele-Referral)) OR (Tele Referral)) OR (Tele-Referrals)) OR (Mobile Health)) OR (Health, Mobile)) OR (mHealth)) OR (Telehealth)) OR (eHealth)) OR (Tele-Intensive Care)) OR (Tele Intensive Care)) OR (Tele-ICU)) OR (Tele ICU)) OR (Telecare)) OR (Tele-Care)) OR (Tele Care) | 162,281 |
| 19 | Tele Care | 65,774 |
| 18 | Tele-Care | 65,774 |
| 17 | Telecare | 65,344 |
| 16 | Tele ICU | 64,611 |
| 15 | Tele-ICU | 64,611 |
| 14 | Tele Intensive Care | 64,669 |
| 13 | Tele-Intensive Care | 64,669 |
| 12 | eHealth | 72,906 |
| 11 | Telehealth | 71,117 |
| 10 | mHealth | 73,107 |
| 9 | Health, Mobile | 116,076 |
| 8 | Mobile Health | 116,076 |
| 7 | Tele-Referrals | 64,582 |
| 6 | Tele Referral | 64,643 |
| 5 | Tele-Referral | 64,643 |
| 4 | Medicine, Virtual | 94,938 |
| 3 | Virtual Medicine | 94,938 |
| 2 | Telemedicine[MeSH Terms] | 49,754 |
| 1 | Parkinson disease[MeSH Terms] | 87,750 |

# Section S2: Outcomes

Motor symptoms: such as unified Parkinson’s disease rating scale (UPDRS) ^1^ and the Movement Disability Society (MDS) released in 2007 the revised MDS-UPDRS based on the UPDRS.^2^ These scales are made up of these sections: Part I: evaluation of mentation, behavior, and mood; Part II: self-evaluation of the activities of daily life (ADLs) including speech, swallowing, handwriting, dressing, hygiene, falling, salivating, turning in bed, walking, and cutting food; Part III: clinician-scored monitored motor evaluation; Part IV: complications of therapy. Both scales that was developed as effort to incorporate elements from existing scales to provide comprehensive but efficient and flexible means to monitor Parkinson’ s disease (PD)-related disability and impairment. Due to the low UPDRS total reported in the included literature, we only selected UPDRS Ⅲ for motor symptoms.

Parkinson’s disease is a neuro-degenerative disease with early prominent death of dopaminergic neurons in the substantia nigra pars compacta (SNpc). The lack of dopamine produced in the basal ganglia can cause movement disorders, which are characterized by classic Parkinson's disease motor symptoms. Parkinson's disease is also associated with many non-motor symptoms, some of which have existed for more than a decade before motor dysfunction. We use the changes in gait and balance ability before and after interventional physical activity as the outcomes of the effect of various types of physical activity on motor symptoms. The Berg Balance Scale (BBS) was used as the most prominent balance test battery. BBS is a functional scale assessing static and dynamic postural control associated with 14 different activities.^3^ It has been validated for patients with PD.^4^ In addition, Balance Evaluation Systems Test (BESTest), Mini-Balance Evaluation Systems Test (Mini-BESTest), Tinetti assessment scale, and Fullerton Advanced Balance, are often used as a scale to evaluate the balance in PD, when BBS was not reported in the included literature, we used these as a surrogate outcome for balance. Gait outcomes: (a) using the speed or time of walking test as an objective outcome to evaluate PD gait (e.g., 10 m walking test); (b) using the 6 min walking test to evaluate walking distance; (c) and time up and go test. In addition, we evaluate non-motor symptoms from four directions: depression (e.g., Beck Depression Inventory, Geriatric Depression Scale, and hospital anxiety and depression scale-depression etc.), anxiety (e.g., Beck anxiety inventory, Geriatric Anxiety Inventory, and hospital anxiety and depression scale-anxiety etc.), and cognition (e.g., Montreal Cognitive Assessment (MOCA), PDQ-39 cognition, Frontal assessment battery, and Color Trails Test etc.). We use the falls efficacy scale and activities-specific balance confidence scale as a subjective indicator to assess concern of falling.^5^ Finally, we also assessed quality of life (e.g., PDQ-8 or 39, or SF-39).

**Reference**

1. Ramaker C, Marinus J, Stiggelbout AM, Van Hilten BJ. Systematic evaluation of rating scales for impairment and disability in Parkinson's disease. *Mov Disord* 2002; **17**(5): 867-76.

2. Goetz CG, Fahn S, Martinez-Martin P, et al. Movement Disorder Society-sponsored revision of the Unified Parkinson's Disease Rating Scale (MDS-UPDRS): Process, format, and clinimetric testing plan. *Mov Disord* 2007; **22**(1): 41-7.

3. Berg KO, Maki BE, Williams JI, Holliday PJ, Wood-Dauphinee SL. Clinical and laboratory measures of postural balance in an elderly population. *Arch Phys Med Rehabil* 1992; **73**(11): 1073-80.

4. Qutubuddin AA, Pegg PO, Cifu DX, Brown R, McNamee S, Carne W. Validating the Berg Balance Scale for patients with Parkinson's disease: a key to rehabilitation evaluation. *Arch Phys Med Rehabil* 2005; **86**(4): 789-92.

5. Bloem BR, Marinus J, Almeida Q, et al. Measurement instruments to assess posture, gait, and balance in Parkinson's disease: Critique and recommendations. *Mov Disord* 2016; **31**(9): 1342-55.

# Section S3: Statistical methods in details

## Network meta-analysis

We will use R software gemtc and rjags packages to perform Bayesian network meta-analysis. Using arm-level data and import into the R software in CSV format. The effect size measure for continuous outcomes chooses the standardized mean difference (SMD) or MD of the change score (end-point minus baseline score). The normal likelihood for continuous outcomes. The study effect sizes were then synthesized using a random-effects network meta-analysis model. In addition, we will present the summary SMD or MD, 95% credible intervals (CrIs) for all pairwise comparisons in the league table, and we show the results of comparing the outcomes of each exercise intervention group and the control group in the form of a forest plot. To rank the various treatments for each outcome, we will use the surface under the cumulative ranking curve (SUCRA). In the process of extracting data, if the original study reported a standard error in the experimental and control groups, the standard deviation was calculated by the formula: standard deviation (SD) = standard error (SE) × √n. If both are missing, we will estimate SD based on the confidence interval, t-value, quartile, range, or p-values as described in section 7.7.3 of the Cochrane Handbook for Systematic Reviews. When only figures were presented, data were extracted using GetData (http://getdata-graph-digitizer.com) to measure the length (in pixels) of the axes to calibrate and then the length in pixels from the relevant axis to the data points of interest. If the data needed for the study cannot be extracted from the above methods, we will ask the authors about the data at least 4 times within 6 weeks.

## Continuous variable-random effects model:

For any physical activity intervention x in any randomized controlled experiment i, the sample size is n_i, x_. The effect of treatment is y_i, x_ (change from baseline), and standard error is se_i, x_. Then the normal

likelihood is employed to y_i, x_ ~ N (𝜃_i, y_, se_i, x_) in each arm. In addition, for any randomized controlled experiment, there should be a basic physical activity type b(i), and its effect is represented by y_i, b(i)_

y_i, b(i)_ = 𝑢_𝑖_

In the random effects model, for any interventional physical activity k that is not a basic physical activity type, its exercise effect is:

𝜃_i, k_= 𝑢_𝑖_+𝛿_i, b(i), k_ for k ≥ 2

Where 𝛿_i, b(i), k_ is the difference between the effect of physical activity k and basic physical activity b, and conforms to the following normal distribution 𝛿_i, x, y_ ~ N(d_x,y_, 𝞼^2^_x, y_), where d_x,y_ is the relative effect of physical activity intervention y and x, 𝞼^2^_x, y_ is the variance of the relative effect of physical activity intervention y and x. In addition, this study presented the final treatment effect with standardised mean difference (SMD), so the above formula is modified:

y_i, b(i)_ = 𝑢_𝑖_/S_i_

𝜃_i, k_= (𝑢_𝑖_+𝛿_i, b(i), y_)/ S_i_ for k ≥ 2

where S_i_ is the pooled standard deviation in the study arms.

model {

# Likelihood for arm-based data

## OMITTED

# Likelihood for contrast-based data (univariate for 2-arm trials)

for(i in studies.r2) {

for (k in 2:na[i]) {

mest[i, k] <- delta[i, k]

}

m[i, 2] ~ dnorm(mest[i, 2], prec[i, 2])

prec[i, 2] <- 1 / (e[i, 2] * e[i, 2])

dev[i, 1] <- pow(m[i, 2] - mest[i, 2], 2) * prec[i, 2]

}

# Likelihood for contrast-based data (multivariate for multi-arm trials)

for(i in studies.rm) {

for (k in 2:na[i]) {

mest[i, k] <- delta[i, k]

}

for (k in 1:(na[i]-1)) {

for (j in 1:(na[i]-1)) {

Sigma[i,j,k] <- ifelse(equals(j, k), pow(e[i,k+1], 2), pow(e[i,1], 2))

}

}

Omega[i,1:(na[i]-1),1:(na[i]-1)] <- inverse(Sigma[i,1:(na[i]-1),1:(na[i]-1)])

m[i,2:na[i]] ~ dmnorm(mest[i,2:na[i]], Omega[i,1:(na[i]-1),1:(na[i]-1)])

mdiff[i, 2:na[i]] <- m[i, 2:na[i]] - mest[i, 2:na[i]]

dev[i, 1] <- t(mdiff[i, 2:na[i]]) %*% Omega[i, 1:(na[i]-1),1:(na[i]-1)] %*% mdiff[i, 2:na[i]]

}

# Random effects model

for (i in studies) {

# Study-level relative effects

w[i, 1] <- 0

delta[i, 1] <- 0

for (k in 2:na[i]) { # parameterize multi-arm trials using a trick to avoid dmnorm

delta[i, k] ~ dnorm(md[i, k], taud[i, k])

md[i, k] <- d[t[i, 1], t[i, k]] + sw[i, k]

taud[i, k] <- tau.d * 2 * (k - 1) / k

w[i, k] <- delta[i, k] - (d[t[i, 1], t[i, k]])

sw[i, k] <- sum(w[i, 1:(k-1)]) / (k - 1)

}

}

# Random effects variance prior

sd.d ~ dunif(0, om.scale)

tau.d <- pow(sd.d, -2)

## Assessment of the transitivity assumption

Different clinical trials need to ensure that their baseline levels are consistent. If the baseline levels are inconsistent, the results cannot be transitive. Therefore, the transitivity assumption was evaluated by comparing the distribution of potential effect modifiers (publication year, sample size, mean age, percentage male, years of diagnosis, and disease grade) across studies grouped before analyzing the results.

## Assessment of heterogeneity and inconsistency

We use the tau square (τ^2^) test and p-value to qualitatively analyze the statistical heterogeneity between the studies. The larger the τ^2^ and the smaller the p-value, the greater the possibility of heterogeneity; on the contrary, the smaller the existence heterogeneity. In addition, I^2^ is a parameter for quantitative analysis of the heterogeneity between the results of each study. It’ s value is distributed from 0-100%. When I^2^ is less than 25%, it means that the heterogeneity is low; 25%-50% means that the heterogeneity is moderate; I^2^ > 75% means high heterogeneity. In summary, when I^2^ > 50%, it means that there is substantial heterogeneity. We will use global and local methods to test the inconsistency of the research results. For global inconsistency, we evaluated inconsistency statistically using the design-by-treatment test. In addition, we will assessment of local inconsistency by separating indirect from direct evidence (SIDE test) using the R netmeta package.

## Assessment of sensitivity

We assessed the sensitivity of our findings by repeating each network meta-analysis after excluding studies at overall high risk of bias.

## Statistical software

The analysis and presentation of results will be performed using the Stata packages network, the R package rjags, gemtc, netmeta, ggplot2, and forestplot.

# Section S4: Assessment of the transitivity

Different clinical trials need to ensure that their baseline levels are consistent. If the baseline levels are inconsistent, the results cannot be transitive. Therefore, the transitivity assumption was evaluated by comparing the distribution of potential effect modifiers (mean age, sample size, percentage male, years of diagnosis, disease grade and specific intervention variables) across studies grouped before analyzing the results, and we use the R ggplot2 package to draw boxplots between the above potential influencing factors and various types of physical activity.

## 4.1 Sample size

We checked the sample size distribution of the included studies. The range is from 4 to 107 in each arm, with a median of 18. The results of one-way ANOVA showed that there was no statistical difference in the sample size between the types of eHealth (F = 0.383, P = 0.819).

## 4.2 Mean age

We checked the mean age distribution of the included study participants. The range is from 57.87 to 72.1, with a median of 65.4. The results of one-way ANOVA showed that there was no statistical difference in the mean age between the types of eHealth (F = 1.251, P = 0.304).

## 4.3 Years of diagnosis

We checked the year of diagnosis distribution of the included study participants. The range is from 3 to 11.67, with a median of 5.9. The results of one-way ANOVA showed that there was no statistical difference in the years of diagnosis between the types of eHealth (F = 0.351, P = 0.841).

## 4.4 Hoehn and Yahr stage

We checked the Hoehn and Yahr stage distribution of the included study participants. The range is from 1.9 to 3, with a median of 2.3. The results of one-way ANOVA showed that there was no statistical difference in the Hoehn and Yahr stage between the types of eHealth (F = 0.309, P = 0.354).

******

## 4.5 Percentage male

We checked the percentage male distribution of the included study participants. The range is from 25 to 100%, with a median of 63.8%. The results of one-way ANOVA showed that there was no statistical difference in the percentage male between the types of eHealth (F = 0.251, P = 0.907).

## 4.6 Intervention period

We checked the intervention period distribution of the included study. The range is from 3 to 48, with a median of 10. The results of one-way ANOVA showed that there was statistical difference in the intervention period between the types of eHealth (F = 5.682, P = 0.000951).

## 4.7 Intervention frequency

We checked the intervention frequency distribution of the included study. The range is from 1 to 7, with a median of 3. The results of one-way ANOVA showed that there no was statistical difference in the intervention frequency between the types of eHealth (F = 0.075, P = 0.989).

## 4.8 Total number of interventions

We checked the total number of intervention distribution of the included study. The range is from 9 to 112, with a median of 18. The results of one-way ANOVA showed that there no was statistical difference in the total number of intervention between the types of eHealth (F = 1.399, P = 0.257).

# Section S5: Network plot and league table

## 5.1 Motor symptoms (UPDRS 3)

The size of the dots represents the sample size, and the thickness of the line represents the number of studies that directly compared different types of mHealth, with specific numbers given.

| **eExercise**  **(0.91)** | -0.15 (-0.91; 0.62) | NA | **-2.38 (-3.36; -1.39)** | NA |
| --- | --- | --- | --- | --- |
| -0.15 (-0.91; 0.62) | **aCG**  **(0.84)** | NA | NA | NA |
| **-2.01 (-3.16; -0.87)** | **-1.87 (-3.25; -0.49)** | **eVisits**  **(0.42)** | -0.36 (-0.95; 0.23) | NA |
| **-2.38 (-3.36; -1.39)** | **-2.23 (-3.48; -0.98)** | -0.36 (-0.95; 0.23) | **UC**  **(0.19)** | -0.25 (-1.59; 1.09) |
| **-2.62 (-4.29; -0.96)** | **-2.48 (-4.31; -0.64)** | -0.61 (-2.07; 0.85) | -0.25 (-1.59; 1.09) | **eCognitive**  **(0.14)** |

All results are presented in the form of SMD (95% CrI). Treatment types are ranked according to the SUCRA for UPDRS 3 with the best from left to right. The results of the network meta-analysis are showed in the lower left part, and results from pairwise comparisons in the upper right half (if available). Cells shown in bold indicate significant results. NA not available, SMD standardized mean difference, CrI Credible Interval.

## 5.2 Quality of life

| **aCG**  **(0.73)** | 0.00 (-0.20; 0.21) | 0.08 (-0.66; 0.82) | NA | NA |
| --- | --- | --- | --- | --- |
| 0.00 (-0.20; 0.21) | **eExercise**  **(0.72)** | NA | NA | NA |
| 0.08 (-0.66; 0.82) | 0.07 (-0.70; 0.84) | **eCognitive**  **(0.71)** | **0.51 ( 0.14; 0.88)** | NA |
| 0.59 (-0.24; 1.42) | 0.58 (-0.27; 1.44) | **0.51 ( 0.14; 0.88)** | **UC**  **(0.18))** | 0.01 (-0.18; 0.20) |
| 0.60 (-0.25; 1.45) | 0.60 (-0.28; 1.47) | **0.52 ( 0.11; 0.94)** | 0.01 (-0.18; 0.20) | **eVisits**  **(0.16)** |

All results are presented in the form of SMD (95% CrI). Treatment types are ranked according to the SUCRA for quality of life with the best from left to right. The results of the network meta-analysis are showed in the lower left part, and results from pairwise comparisons in the upper right half (if available). Cells shown in bold indicate significant results. NA not available, SMD standardized mean difference, CrI Credible Interval.

## 5.3 Cognitive level

| **eCognitive**  **(0.91)** | NA | NA | **0.95 ( 0.06; 1.84)** | **1.09 ( 0.18; 2.00)** |
| --- | --- | --- | --- | --- |
| 0.27 (-0.84; 1.38) | **eExercise**  **(0.74)** | NA | **0.68 ( 0.02; 1.34)** | NA |
| 0.92 (-0.04; 1.87) | 0.64 (-0.82; 2.11) | **eVisits**  **(0.41)** | NA | 0.18 (-0.11; 0.46) |
| **0.95 ( 0.06; 1.84)** | **0.68 ( 0.02; 1.34)** | 0.03 (-1.27; 1.34) | **aCG**  **(0.28)** | NA |
| **1.09 ( 0.18; 2.00)** | 0.82 (-0.62; 2.25) | 0.18 (-0.11; 0.46) | 0.14 (-1.14; 1.42) | **UC**  **(0.17)** |

All results are presented in the form of SMD (95% CrI). Treatment types are ranked according to the SUCRA for cognitive level with the best from left to right. The results of the network meta-analysis are showed in the lower left part, and results from pairwise comparisons in the upper right half (if available). Cells shown in bold indicate significant results. NA not available, SMD standardized mean difference, CrI Credible Interval.

## 5.4 Depressive Symptoms

| **eCognitive**  **(1.0)** | NA | **-1.16 (-1.63; -0.69)** | NA | **-1.40 (-1.90; -0.91)** |
| --- | --- | --- | --- | --- |
| **-1.12 (-1.75; -0.49)** | **eExercise**  **(0.52)** | -0.04 (-0.46; 0.38) | NA | NA |
| **-1.16 (-1.63; -0.69)** | -0.04 (-0.46; 0.38) | **aCG**  **(0.48))** | NA | NA |
| **-1.39 (-1.94; -0.85)** | -0.27 (-1.11; 0.56) | -0.23 (-0.95; 0.48) | **eVisits**  **(0.26)** | -0.01 (-0.24; 0.23) |
| **-1.40 (-1.90; -0.91)** | -0.28 (-1.08; 0.51) | -0.24 (-0.92; 0.44) | -0.01 (-0.24; 0.23) | **UC**  **(0.24)** |

All results are presented in the form of SMD (95% CrI). Treatment types are ranked according to the SUCRA for depressive symptoms with the best from left to right. The results of the network meta-analysis are showed in the lower left part, and results from pairwise comparisons in the upper right half (if available). Cells shown in bold indicate significant results. NA not available, SMD standardized mean difference, CrI Credible Interval.

## 5.5 Anxiety Symptoms

| **eCognitive**  **(1.0)** | NA | **-0.93 (-1.43; -0.44)** | NA | **-1.18 (-1.61; -0.75)** |
| --- | --- | --- | --- | --- |
| **-0.90 (-1.50; -0.29)** | **eExercise**  **(0.51)** | -0.04 (-0.39; 0.31) | NA | NA |
| **-0.93 (-1.43; -0.44)** | -0.04 (-0.39; 0.31) | **aCG**  **(0.46)** | NA | NA |
| **-1.09 (-1.63; -0.55)** | -0.19 (-1.00; 0.62) | -0.15 (-0.88; 0.58) | **eVisits**  **(0.36)** | -0.09 (-0.42; 0.23) |
| **-1.18 (-1.61; -0.75)** | -0.28 (-1.03; 0.46) | -0.25 (-0.90; 0.41) | -0.09 (-0.42; 0.23) | **UC**  **(0.19)** |

All results are presented in the form of SMD (95% CrI). Treatment types are ranked according to the SUCRA for anxiety symptoms with the best from left to right. The results of the network meta-analysis are showed in the lower left part, and results from pairwise comparisons in the upper right half (if available). Cells shown in bold indicate significant results. NA not available, SMD standardized mean difference, CrI Credible Interval.

# Section S6: Evaluation of heterogeneity and inconsistency

We use the tau square (τ^2^) test and p-value to qualitatively analyze the statistical heterogeneity between the studies. The larger the τ^2^ and the smaller the p-value, the greater the possibility of heterogeneity; on the contrary, the smaller the existence heterogeneity. In addition, I^2^ is a parameter for quantitative analysis of the heterogeneity between the results of each study. It’ s value is distributed from 0-100%. When I^2^ is less than 25%, it means that the heterogeneity is low; 25%-50% means that the heterogeneity is moderate; I^2^ > 75% means high heterogeneity. In summary, when I^2^ > 50%, it means that there is substantial heterogeneity.

| Outcomes | Quantifying heterogeneity | | | | | | Global inconsistency |
| --- | --- | --- | --- | --- | --- | --- | --- |
|  | τ^2^ | Q | df | P | I^2^ | Heterogeneity  assessment | The Design-by-Treatment test |
| Motor symptoms | 0.1000 | 13.71 | 8 | 0.0565 | 49% | Moderate | 0.5437 |
| Quality of life | 0 | 10.12 | 12 | 0.6058 | 0% | Low | 0.9661 |
| Cognitive level | 0 | 0.23 | 1 | 0.6305 | 0% | Low | 0.4234 |
| Depressive symptoms | 0.0146 | 7.18 | 6 | 0.3048 | 16.4% | Low | 0.8149 |
| Anxiety symptoms | 0.0011 | 1 | 1 | 0.3163 | 0.4% | Low | 0.8355 |

# Section S7: Sensitivity analysis

We assessed the sensitivity of our findings by repeating each pairwise and network meta-analysis after excluding studies at overall high risk of bias (Lakshminarayana et al. (2017), and Wilkinson et al. (2016)). Since the included high-risk literature did not evaluate cognition, falls, 6-minute walk test, TUG, walking speed, and balance ability, we did not re-analyze these indicators.

## 7.1 Sensitivity analysis for pairwise meta-analysis

Figure 7.1.1 Forest plot of UPDRS-3 pairwise meta-analysis. After excluding high risk (Wilkinson et al. (2016)), our results still showed that telemedicine significantly alleviate overall motor symptoms in patients with Parkinson's disease.

Figure 7.1.2 Forest plot of pairwise meta-analysis of quality of life. After excluding high risk (Lakshminarayana et al. (2017), and Wilkinson et al. (2016)), our results still showed that telemedicine did not significantly improve quality of life in patients with Parkinson's disease.

Figure 7.1.3 Forest plot of pairwise meta-analysis of depressive symptoms. After excluding high risk (Lakshminarayana et al. (2017), and Wilkinson et al. (2016)), our results still showed that telemedicine significantly alleviate depressive symptoms in patients with Parkinson's disease.

Figure 7.1.4 Forest plot of pairwise meta-analysis of anxiety symptoms. After excluding high risk (Lakshminarayana et al. (2017)), our results still showed that telemedicine significantly alleviate anxiety symptoms in patients with Parkinson's disease.

## 7.2 Sensitivity analysis for network meta-analysis

| **eExercise**  **(0.91)** | -0.15 (-0.89; 0.60) | NA | **-2.38 (-3.34; -1.42)** | NA |
| --- | --- | --- | --- | --- |
| -0.15 (-0.89; 0.60) | **aCG**  **(0.84)** | NA | NA | NA |
| **-2.18 (-3.33; -1.03)** | **-2.03 (-3.40; -0.67)** | **eVisits**  **(0.37)** | -0.20 (-0.83; 0.43) | NA |
| **-2.38 (-3.34; -1.42)** | **-2.23 (-3.45; -1.02)** | -0.20 (-0.83; 0.43) | **UC**  **(0.23)** | -0.25 (-1.55; 1.05) |
| **-2.63 (-4.24; -1.01)** | **-2.48 (-4.26; -0.70)** | -0.45 (-1.89; 1.00) | -0.25 (-1.55; 1.05) | **eCognitive**  **(0.16)** |

All results are presented in the form of SMD (95% CrI). Treatment types are ranked according to the SUCRA for UPDRS 3 with the best from left to right. The results of the network meta-analysis are showed in the lower left part, and results from pairwise comparisons in the upper right half (if available). Cells shown in bold indicate significant results. NA not available, SMD standardized mean difference, CrI Credible Interval. After excluding high risk (Wilkinson et al. (2016)), our results still showed that only eExercise and aCG significantly alleviate overall motor symptoms in patients with Parkinson's disease.

| **aCG**  **(0.74)** | 0.00 (-0.20; 0.21) | 0.08 (-0.66; 0.82) | NA | NA |
| --- | --- | --- | --- | --- |
| 0.00 (-0.20; 0.21) | **eExercise**  **(0.72)** | NA | NA | NA |
| 0.08 (-0.66; 0.82) | 0.07 (-0.70; 0.84) | **eCognitive**  **(0.71)** | **0.51 ( 0.14; 0.88)** | NA |
| 0.59 (-0.24; 1.42) | 0.58 (-0.27; 1.44) | **0.51 ( 0.14; 0.88)** | **UC**  **(0.18)** | 0.01 (-0.25; 0.26) |
| 0.60 (-0.27; 1.46) | 0.59 (-0.30; 1.48) | **0.52 ( 0.07; 0.97)** | 0.01 (-0.25; 0.26) | **eVisits**  **(0.17))** |

All results are presented in the form of SMD (95% CrI). Treatment types are ranked according to the SUCRA for quality of life with the best from left to right. The results of the network meta-analysis are showed in the lower left part, and results from pairwise comparisons in the upper right half (if available). Cells shown in bold indicate significant results. NA not available, SMD standardized mean difference, CrI Credible Interval. After excluding high risk (Lakshminarayana et al. (2017), and Wilkinson et al. (2016)), our results still showed that only eCognitivie significantly improve quality of life in patients with Parkinson's disease.

| **eCognitive**  **(1.0)** | NA | **-1.10 (-1.71; -0.49)** | NA | **-1.33 (-2.01; -0.66)** |
| --- | --- | --- | --- | --- |
| **-1.06 (-1.97; -0.15)** | **eExercise**  **(0.47)** | -0.04 (-0.72; 0.64) | NA | NA |
| **-1.10 (-1.71; -0.49)** | -0.04 (-0.72; 0.64) | **aCG**  **(0.45)** | NA | NA |
| **-1.31 (-2.14; -0.48)** | -0.25 (-1.49; 0.98) | -0.21 (-1.24; 0.82) | **eVisits**  **(0.31)** | -0.02 (-0.51; 0.47) |
| **-1.33 (-2.01; -0.66)** | -0.27 (-1.41; 0.86) | -0.23 (-1.14; 0.67) | -0.02 (-0.51; 0.47) | **UC**  **(0.27)** |

All results are presented in the form of SMD (95% CrI). Treatment types are ranked according to the SUCRA for depressive symptoms with the best from left to right. The results of the network meta-analysis are showed in the lower left part, and results from pairwise comparisons in the upper right half (if available). Cells shown in bold indicate significant results. NA not available, SMD standardized mean difference, CrI Credible Interval. After excluding high risk (Lakshminarayana et al. (2017), and Wilkinson et al. (2016)), our results still showed that eCognitive significantly alleviate depressive symptoms in patients with Parkinson's disease.

| **eCognitive**  **(1.0)** | NA | **-0.93 (-1.43; -0.44)** | **-1.18 (-1.61; -0.75)** |
| --- | --- | --- | --- |
| **-0.90 (-1.50; -0.29)** | **eExercise**  **(0.45)** | -0.04 (-0.39; 0.31) | NA |
| **-0.93 (-1.43; -0.44)** | -0.04 (-0.39; 0.31) | **aCG**  **(0.40)** | NA |
| **-1.18 (-1.61; -0.75)** | -0.28 (-1.03; 0.46) | -0.25 (-0.90; 0.41) | **UC**  **(0.15)** |

All results are presented in the form of SMD (95% CrI). Treatment types are ranked according to the SUCRA for anxiety symptoms with the best from left to right. The results of the network meta-analysis are showed in the lower left part, and results from pairwise comparisons in the upper right half (if available). Cells shown in bold indicate significant results. NA not available, SMD standardized mean difference, CrI Credible Interval. After excluding high risk (Lakshminarayana et al. (2017)), our results still showed that eCognitive significantly alleviate anxiety symptoms in patients with Parkinson's disease.

# Section S8: Pairwise meta-analysis

Figure 8.1 Forest plot of telemedicine on total motor symptoms in PD patients.

Figure 8.2 Forest plot of telemedicine on quality of life in PD patients.

Figure 8.3 Forest plot of telemedicine on cognitive level in PD patients.

Figure 8.4 Forest plot of telemedicine on depressive symptoms in PD patients.

Figure 8.5 Forest plot of telemedicine on anxiety symptoms in PD patients.

Figure 8.6 Forest plot of telemedicine on fear of falling in PD patients.

Figure 8.7 Forest plot of telemedicine on 6 min walk test in PD patients.

Figure 8.8 Forest plot of telemedicine on walking velocity in PD patients.

Figure 8.9 Forest plot of telemedicine on balance ability in PD patients.

Figure 8.10 Forest plot of telemedicine on TUG in PD patients.

# Section S9: Evaluation outcome measurement method included in the literature

| **Study** | **Motor symptom** | **Quality of life** | **Cognitive level** | **Depression** | **Anxiety** | **6 min walk test** | **TUG** | **Walking velocity** | **Mini-BEST** | **Number of falls** |
| --- | --- | --- | --- | --- | --- | --- | --- | --- | --- | --- |
| Park, Kim [1] | Unifed Parkinson's disease Rating Scale (UPDRS) part III (motor) | Parkinson's disease Questionnaire-39 | NA | NA | NA | NA | NA | NA | NA | NA |
| Maggio, Luca [2] | NA | NA | Montreal Cognitive Assessment | Hamilton Depression Rating Scale | NA | NA | NA | NA | NA | NA |
| Eldemir, Guclu-Gunduz [3] | Unifed Parkinson's disease Rating Scale (UPDRS) part III (motor) | Parkinson's disease Questionnaire-8 | NA | NA | NA | NA | NA | NA | NA | NA |
| Wuthrich and Rapee [4] | NA | NA | NA | 42 item Depression, Anxiety and Stress Scales (Depression) | 42 item Depression, Anxiety and Stress Scales (Anxiety) | NA | NA | NA | NA | NA |
| van der Kolk, de Vries [5] | the Movement Disorders Society—Unified Parkinson’s Disease Rating Scale (motor) | Parkinson's disease Questionnaire-39 | Montreal Cognitive Assessment | Hospital Anxiety and Depression Scale depression score | Hospital Anxiety and Depression Scale anxiety score | 6 min walk test (m) | Timed Up and Go test (s) | NA | Mini-Balance Evaluation Systems Test | Number of falls |
| Kaya Aytutuldu, Ersoz Huseyinsinoglu [6] | NA | Parkinson's disease Questionnaire-39 | NA | NA | NA | NA | Timed Up and Go test (s) | 3 m walking test (m/s) | Mini-Balance Evaluation Systems Test | Activity-Specific Balance Confidence Scale Short Form |
| Gandolfi, Geroin [7] | NA | Parkinson's disease Questionnaire-8 | NA | NA | NA | NA | NA | 10 m walking test (m/s) | Berg Balance Scale | Number of falls |
| Vasconcellos, Silva [8] | NA | NA | NA | NA | NA | NA | NA | 8 m walking test (m/s) | NA | NA |
| Dobkin, Mann [9] | NA | NA | NA | Hospital Anxiety and Depression Scale depression score | Hospital Anxiety and Depression Scale anxiety score | NA | NA | NA | NA | NA |
| Goffredo, Baglio [10] | the Movement Disorders Society—Unified Parkinson’s Disease Rating Scale (motor) | NA | NA | NA | NA | 6 min walk test (m) | Timed Up and Go test (s) | mini-BESTest dynamic walking | Mini-Balance Evaluation Systems Test | NA |
| Ginis, Nieuwboer [11] | the Movement Disorders Society—Unified Parkinson’s Disease Rating Scale (motor) | the Short Form 36 Health Survey | Color Trail Test A and B and verbal uency scores in sitting and walking | NA | NA | 2 min walk test (m) | NA | Gait speed (m/s) | Mini-Balance Evaluation Systems Test | the Falls Efficacy Scale-International |
| Lai, Bond [12] | NA | NA | NA | NA | NA | 6 min walk test (m) | NA | 10 m walking test (m/s) | NA | NA |
| Dorsey, Deuel [13] | Unifed Parkinson's disease Rating Scale (UPDRS) part III (motor) | Parkinson's disease Questionnaire-39 | Montreal Cognitive Assessment | Geriatric Depression Scale 15 | NA | NA | NA | NA | NA | NA |
| Beck, Beran [14] | the Movement Disorders Society—Unified Parkinson’s Disease Rating Scale (motor) | Parkinson's disease Questionnaire-39 | Montreal Cognitive Assessment | Geriatric Depression Scale 15 | NA | NA | NA | NA | NA | NA |
| Dobkin, Mann [15] | NA | Quality of life | NA | Hospital Anxiety and Depression Scale depression score | Hospital Anxiety and Depression Scale anxiety score | NA | NA | NA | NA | NA |
| Ellis, Cavanaugh [16] | NA | Parkinson's disease Questionnaire-39 | NA | NA | NA | 6 min walk test (m) | NA | NA | NA | NA |
| Patel, Ojo [17] | NA | Parkinson's disease Questionnaire-8 | NA | Patient Health Questionnaire | NA | NA | NA | NA | NA | NA |
| Lakshminarayana, Wang [18] | NA | Parkinson's disease Questionnaire-39 | NA | Hospital Anxiety and Depression Scale depression score | Hospital Anxiety and Depression Scale anxiety score | NA | NA | NA | NA | NA |
| Heldman, Harris [19] | Unifed Parkinson's disease Rating Scale (UPDRS) part III (motor) | Parkinson's disease Questionnaire-39 | NA | NA | NA | NA | NA | NA | NA | NA |
| Cubo, Mariscal [20] | Unifed Parkinson's disease Rating Scale (UPDRS) part III (motor) | Quality-adjusted life-years | NA | Hospital Anxiety and Depression Scale depression score | NA | NA | NA | NA | NA | NA |
| Dorsey, Venkataraman [21] | Unifed Parkinson's disease Rating Scale (UPDRS) part III (motor) | Parkinson's disease Questionnaire-39 | NA | NA | NA | NA | NA | NA | NA | NA |
| Khalil, Busse [22] | the Movement Disorders Society—Unified Parkinson’s Disease Rating Scale (motor) | NA | NA | NA | NA | 6 min walk test (m) | NA | 10 m walking test (m/s) | Mini-Balance Evaluation Systems Test | the Falls Efficacy Scale-International |
| Wilkinson, Spindler [23] | Unifed Parkinson's disease Rating Scale (UPDRS) part III (motor) | Parkinson's disease Questionnaire-39 | NA | Geriatric Depression Scale 15 | NA | NA | NA | NA | NA | NA |

1. Park Y, Kim SR, So HY, Jo S, Lee SH, Hwang YS, et al. Effect of mobile health intervention for self-management on self-efficacy, motor and non-motor symptoms, self-management, and quality of life in people with Parkinson's disease: Randomized controlled trial. Geriatr Nurs. 2022;46:90-7. [doi: 10.1016/j.gerinurse.2022.05.003] [Medline: 35643018].

2. Maggio MG, Luca A, Cicero CE, Calabrò RS, Drago F, Zappia M, et al. Effectiveness of telerehabilitation plus virtual reality (Tele-RV) in cognitive e social functioning: A randomized clinical study on Parkinson's disease. Parkinsonism Relat Disord. 2024;119:105970. [doi: 10.1016/j.parkreldis.2023.105970] [Medline: 38142630].

3. Eldemir S, Guclu-Gunduz A, Eldemir K, Saygili F, Yilmaz R, Akbostancı MC. The effect of task-oriented circuit training-based telerehabilitation on upper extremity motor functions in patients with Parkinson's disease: A randomized controlled trial. Parkinsonism Relat Disord. 2023;109:105334. [doi: 10.1016/j.parkreldis.2023.105334] [Medline: 36917914].

4. Wuthrich VM, Rapee RM. Telephone-Delivered Cognitive Behavioural Therapy for Treating Symptoms of Anxiety and Depression in Parkinson's Disease: A Pilot Trial. Clin Gerontol. 2019;42(4):444-53. [doi: 10.1080/07317115.2019.1580811] [Medline: 30821649].

5. van der Kolk NM, de Vries NM, Kessels RPC, Joosten H, Zwinderman AH, Post B, et al. Effectiveness of home-based and remotely supervised aerobic exercise in Parkinson's disease: a double-blind, randomised controlled trial. Lancet Neurol. 2019;18(11). [doi: 10.1016/S1474-4422(19)30285-6] [Medline: 31521532].

6. Kaya Aytutuldu G, Ersoz Huseyinsinoglu B, Karagoz Sakalli N, Sen A, Yeldan I. LSVT® BIG versus progressive structured mobility training through synchronous telerehabilitation in Parkinson's disease: A randomized controlled trial. Neurol Sci. 2024;45(7):3163-72. [doi: 10.1007/s10072-024-07322-0] [Medline: 38267603].

7. Gandolfi M, Geroin C, Dimitrova E, Boldrini P, Waldner A, Bonadiman S, et al. Virtual Reality Telerehabilitation for Postural Instability in Parkinson's Disease: A Multicenter, Single-Blind, Randomized, Controlled Trial. Biomed Res Int. 2017;2017:7962826. [doi: 10.1155/2017/7962826] [Medline: 29333454].

8. Vasconcellos LSd, Silva RS, Pachêco TB, Nagem DA, Sousa CdO, Ribeiro TS. Telerehabilitation-based trunk exercise training for motor symptoms of individuals with Parkinson's disease: A randomized controlled clinical trial. J Telemed Telecare. 2023;29(9):698-706. [doi: 10.1177/1357633X211021740] [Medline: 34142896].

9. Dobkin RD, Mann SL, Weintraub D, Rodriguez KM, Miller RB, St Hill L, et al. Innovating Parkinson's Care: A Randomized Controlled Trial of Telemedicine Depression Treatment. Mov Disord. 2021;36(11):2549-58. [doi: 10.1002/mds.28548] [Medline: 33710659].

10. Goffredo M, Baglio F, De Icco R, Proietti S, Maggioni G, Turolla A, et al. Efficacy of non-immersive virtual reality-based telerehabilitation on postural stability in Parkinson's disease: a multicenter randomized controlled trial. Eur J Phys Rehabil Med. 2023;59(6):689-96. [doi: 10.23736/S1973-9087.23.07954-6] [Medline: 37847247].

11. Ginis P, Nieuwboer A, Dorfman M, Ferrari A, Gazit E, Canning CG, et al. Feasibility and effects of home-based smartphone-delivered automated feedback training for gait in people with Parkinson's disease: A pilot randomized controlled trial. Parkinsonism Relat Disord. 2016;22:28-34. [Medline: 26777408].

12. Lai B, Bond K, Kim Y, Barstow B, Jovanov E, Bickel CS. Exploring the uptake and implementation of tele-monitored home-exercise programmes in adults with Parkinson's disease: A mixed-methods pilot study. J Telemed Telecare. 2020;26(1-2):53-63. [doi: 10.1177/1357633X18794315] [Medline: 30134777].

13. Dorsey ER, Deuel LM, Voss TS, Finnigan K, George BP, Eason S, et al. Increasing access to specialty care: a pilot, randomized controlled trial of telemedicine for Parkinson's disease. Mov Disord. 2010;25(11):1652-9. [doi: 10.1002/mds.23145] [Medline: 20533449].

14. Beck CA, Beran DB, Biglan KM, Boyd CM, Dorsey ER, Schmidt PN, et al. National randomized controlled trial of virtual house calls for Parkinson disease. Neurology. 2017;89(11):1152-61. [doi: 10.1212/WNL.0000000000004357] [Medline: 28814455].

15. Dobkin RD, Mann SL, Gara MA, Interian A, Rodriguez KM, Menza M. Telephone-based cognitive behavioral therapy for depression in Parkinson disease: A randomized controlled trial. Neurology. 2020;94(16):e1764-e73. [doi: 10.1212/WNL.0000000000009292] [Medline: 32238507].

16. Ellis TD, Cavanaugh JT, DeAngelis T, Hendron K, Thomas CA, Saint-Hilaire M, et al. Comparative Effectiveness of mHealth-Supported Exercise Compared With Exercise Alone for People With Parkinson Disease: Randomized Controlled Pilot Study. Phys Ther. 2019;99(2):203-16. [doi: 10.1093/ptj/pzy131] [Medline: 30715489].

17. Patel S, Ojo O, Genc G, Oravivattanakul S, Huo Y, Rasameesoraj T, et al. A Computerized Cognitive behavioral therapy Randomized, Controlled, pilot trial for insomnia in Parkinson Disease (ACCORD-PD). J Clin Mov Disord. 2017;4:16. [doi: 10.1186/s40734-017-0062-2] [Medline: 28852567].

18. Lakshminarayana R, Wang D, Burn D, Chaudhuri KR, Galtrey C, Guzman NV, et al. Using a smartphone-based self-management platform to support medication adherence and clinical consultation in Parkinson's disease. NPJ Parkinsons Dis. 2017;3:2. [doi: 10.1038/s41531-016-0003-z] [Medline: 28649602].

19. Heldman DA, Harris DA, Felong T, Andrzejewski KL, Dorsey ER, Giuffrida JP, et al. Telehealth Management of Parkinson's Disease Using Wearable Sensors: An Exploratory Study. Digit Biomark. 2017;1(1):43-51. [doi: 10.1159/000475801] [Medline: 29725667].

20. Cubo E, Mariscal N, Solano B, Becerra V, Armesto D, Calvo S, et al. Prospective study on cost-effectiveness of home-based motor assessment in Parkinson's disease. J Telemed Telecare. 2017;23(2):328-38. [doi: 10.1177/1357633X16638971] [Medline: 27000142].

21. Dorsey ER, Venkataraman V, Grana MJ, Bull MT, George BP, Boyd CM, et al. Randomized controlled clinical trial of "virtual house calls" for Parkinson disease. JAMA Neurol. 2013;70(5):565-70. [doi: 10.1001/jamaneurol.2013.123] [Medline: 23479138].

22. Khalil H, Busse M, Quinn L, Nazzal M, Batyha W, Alkhazaleh S, et al. A pilot study of a minimally supervised home exercise and walking program for people with Parkinson's disease in Jordan. Neurodegener Dis Manag. 2017;7(1):73-84. [doi: 10.2217/nmt-2016-0041] [Medline: 28097926].

23. Wilkinson JR, Spindler M, Wood SM, Marcus SC, Weintraub D, Morley JF, et al. High patient satisfaction with telehealth in Parkinson disease: A randomized controlled study. Neurol Clin Pract. 2016;6(3):241-51. [Medline: 27347441].

# Section S10: Network meta-regression

**Table 10.1: Summary of network meta-regression results**

| **Covariate** | **Shared beta**  **(median and 95% CrI)** | | **Heterogeneity**  **τ (median and 95% CrI)** | | **% of variance explained** | |
| --- | --- | --- | --- | --- | --- | --- |
| Total motor symptoms | | | | | |  |
| None | - | 0.36 (0.02; 1.04) | | - | |  |
| Mean Age (years) | -2.31 (-4.22; -0.36)* | 0.13 (0.01; 0.58) | | -87.0% | |  |
| Sample Size (n) | 0.19 (-0.59; 0.99) | 0.42 (0.04; 1.13) | | 36.1% | |  |
| Years of Diagnosis | 0.45 (-1.61; 2.50) | 0.39 (0.02; 1.08) | | 17.4% | |  |
| Hoehn and Yahr stage | -1.14 (-3.19; 0.60) | 0.28 (0.01; 0.94) | | -47.8% | |  |
| Percentage Male (%) | 0.46 (-1.59; 2.49) | 0.38 (0.03; 1.09) | | 11.4% | |  |
| Interventional Period (weeks) | 0.04 (-1.35; 1.47) | 0.42 (0.04; 1.11) | | 36.1% | |  |
| Interventional Frequency | -0.19 (-1.73; 1.19) | 0.43 (0.05; 1.11) | | 42.7% | |  |
| Total intervention times | -0.16 (-1.19; 0.84) | 0.43 (0.04; 1.14) | | 42.7% | |  |
| Quality of life | | | | | |  |
| None | - | 0.07 (0.00; 0.30) | | - | |  |
| Mean Age (years) | -0.22 (-0.24; 0.78) | 0.09 (0.00; 0.35) | | 65.3% | |  |
| Sample Size (n) | -0.11 (-0.46; 0.23) | 0.08 (0.00; 0.34) | | 30.6% | |  |
| Years of Diagnosis | -0.09 (-0.71; 0.55) | 0.09 (0.00; 0.35) | | 65.3% | |  |
| Hoehn and Yahr stage | 0.17 (-0.46; 0.86) | 0.08 (0.00; 0.35) | | 30.6% | |  |
| Percentage Male (%) | 0.52 (-0.12; 1.18) | 0.08 (0.00; 0.33) | | 30.6% | |  |
| Interventional Period (weeks) | -0.04 (-0.60; 0.48) | 0.09 (0.00; 0.37) | | 65.3% | |  |
| Interventional Frequency | -0.04 (-0.37; 0.30) | 0.08 (0.00; 0.35) | | 30.6% | |  |
| Total intervention times | -0.01 (-0.50; 0.50) | 0.08 (0.00; 0.35) | | 30.6% | |  |
| Cognitive level | | | | | |  |
| None | - | 0.41 (0.02; 1.05) | | - | |  |
| Mean Age (years) | -0.22 (-0.24; 0.78) | 0.47 (0.03; 1.05) | | 31.4% | |  |
| Sample Size (n) | -0.15 (-1.49; 1.14) | 0.50 (0.02; 1.06) | | 48.7% | |  |
| Years of Diagnosis | -0.16 (-3.70; 2.65) | 0.42 (0.02; 1.05) | | 4.9% | |  |
| Hoehn and Yahr stage | 0.15 (-1.16; 1.50) | 0.48 (0.02; 1.06) | | 37.1% | |  |
| Percentage Male (%) | 0.15 (-1.01; 1.46) | 0.50 (0.03; 1.05) | | 48.7% | |  |
| Interventional Period (weeks) | -0.12 (-2.29; 1.82) | 0.48 (0.02; 1.06) | | 37.1% | |  |
| Interventional Frequency | 0.15 (-5.54; 13.75) | 0.42 (0.01; 1.04) | | 4.9% | |  |
| Total intervention times | -0.18 (-2.35; 1.71) | 0.47 (0.02; 1.06) | | 31.4% | |  |
| Depressive symptoms | | | | | |  |
| None | - | 0.21 (0.01; 0.83) | | - | |  |
| Mean Age (years) | 0.07 (-0.82; 0.89) | 0.27 (0.01; 1.01) | | 65.3% | |  |
| Sample Size (n) | 0.00 (-0.76; 0.75) | 0.28 (0.01; 1.03) | | 77.8% | |  |
| Years of Diagnosis | 0.20 (-0.74; 1.23) | 0.27 (0.01; 0.98) | | 65.3% | |  |
| Hoehn and Yahr stage | -0.23 (-1.40; 0.90) | 0.22 (0.01; 0.92) | | 9.8% | |  |
| Percentage Male (%) | -0.41 (-1.31; 0.48) | 0.21 (0.01; 0.84) | | 0% | |  |
| Interventional Period (weeks) | 0.13 (-0.87; 1.11) | 0.28 (0.02; 1.02) | | 77.8% | |  |
| Interventional Frequency | -0.05 (-1.03; 0.96) | 0.29 (0.01; 1.05) | | 90.7% | |  |
| Total intervention times | 0.21 (-0.86; 1.24) | 0.24 (0.02; 1.24) | | 30.6% | |  |
| Anxiety symptoms | | | | | |  |
| None | - | 0.55 (0.02; 1.21) | | - | |  |
| Mean Age (years) | 0.43 (-3.00; 6.41) | 0.54 (0.02; 1.21) | | -3.6% | |  |
| Sample Size (n) | -0.47 (-3.85; 1.77) | 0.55 (0.02; 1.21) | | 0% | |  |
| Years of Diagnosis | 0.44 (-2.53; 4.63) | 0.55 (0.02; 1.22) | | 0% | |  |
| Hoehn and Yahr stage | -0.04 (-11.54; 16.97) | 0.54 (0.03; 1.22) | | -3.6% | |  |
| Percentage Male (%) | -0.39 (-2.47; 1.38) | 0.57 (0.03; 1.21) | | 7.4% | |  |
| Interventional Period (weeks) | -0.11 (-17.43; 8.41) | 0.53 (0.03; 1.21) | | -7.1% | |  |
| Interventional Frequency | -0.11 (-5.95; 5.11) | 0.54 (0.03; 1.21) | | -3.6% | |  |
| Total intervention times | -0.15 (-9.34; 8.69) | 0.55 (0.03; 1.21) | | 0% | |  |

**10.2 Subgroup analysis of significant factors**

Our network meta-regression results showed that only age significantly affected the overall motor symptoms of PD patients with telemedicine. Estimates at the centering value: age = 64.86818. The adjusted forest plot results showed that the overall ranking and effect size did not change. At the same time, our results also proved to be robust.
